# Machine intelligence decrypts β-lapachone as an allosteric 5-lipoxygenase inhibitor†
†Electronic supplementary information (ESI) available: Supplementary figures, data and methods. See DOI: 10.1039/c8sc02634c


**DOI:** 10.1039/c8sc02634c

**Published:** 2018-07-17

**Authors:** Tiago Rodrigues, Markus Werner, Jakob Roth, Eduardo H. G. da Cruz, Marta C. Marques, Padma Akkapeddi, Susana A. Lobo, Andreas Koeberle, Francisco Corzana, Eufrânio N. da Silva Júnior, Oliver Werz, Gonçalo J. L. Bernardes

**Affiliations:** a Instituto de Medicina Molecular , Faculdade de Medicina da Universidade de Lisboa , Av Prof Egaz Moniz , 1649-028 Lisboa , Portugal . Email: tiago.rodrigues@medicina.ulisboa.pt ; Email: gbernardes@medicina.ulisboa.pt; b Institute of Pharmacy , Friedrich-Schiller-University Jena , Philosophenweg 14 , D-07743 , Jena , Germany; c Institute of Exact Sciences , Department of Chemistry , Federal University of Minas Gerais , Belo Horizonte , Brazil; d Departamento de Química , Centro de Investigacíon en Síntesis Química , Universidad de la Rioja , 26006 Logroño , Spain; e Department of Chemistry , University of Cambridge , Lensfield Road , CB2 1EW Cambridge , UK . Email: gb453@cam.ac.uk

## Abstract

Using machine learning, targets were identified for β-lapachone. Resorting to biochemical assays, β-lapachone was validated as a potent, ligand efficient, allosteric and reversible modulator of 5-lipoxygenase (5-LO). Moreover, we provide a rationale for 5-LO modulation and show that inhibition of 5-LO is relevant for the anticancer activity of β-lapachone. This work demonstrates the power of machine intelligence to deconvolute complex phenotypes, as an alternative and/or complement to chemoproteomics and as a viable general approach for systems pharmacology studies.

## Abstract 2

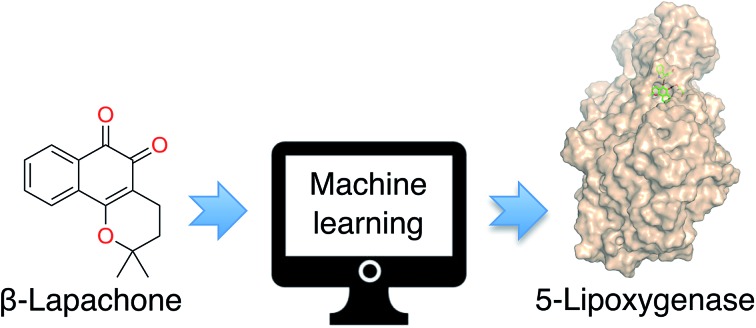
Using machine learning, targets were identified for β-lapachone.

## Introduction

Natural products (NPs) provide inspiration for developing disease-modulating chemical matter and make up *ca.* 40% of all approved drugs,^1^–^3^ but their use in molecular medicine is declining.^4^ Despite progresses in synthetic methodologies to provide scalable routes to NPs, their utility remains hampered by limited knowledge of on-/off-targets that could explain phenotypic effects and liabilities. In this regard, drug target identification remains a daunting task in modern drug discovery, which is often tackled through chemoproteomics.^5^–^9^ Successful chemoproteomics requires tagging of ligands. Apart from the identification of proper tagging sites and potentially extensive synthetic work, ligand derivatization may severely alter the binding affinity to on- and/or off-targets. Therefore, machine intelligence for target identification, which does not suffer from such drawbacks, is of high value.

**Fig. 1:**
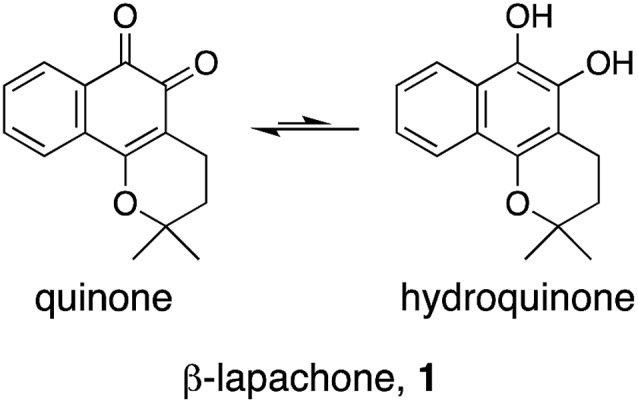
β-Lapachone, **1**, may be present in the quinone or hydroquinone forms.

β-Lapachone (**1**, Fig. 1) is a clinical-stage, natural naphthoquinone with antitumor activity once activated by NAD(P)H:quinone oxireductase 1 (NQO1).^10^ Although bioactivation by NQO1 results in anticancer activity,^11^ it is conceivable that additional proteins are involved in the mode of action of **1**. Discovery of those proteins can constitute an important hallmark in the understanding of pharmacology networks for **1**. Additionally, it may leverage the development of β-lapachone-based drug delivery systems to mask the much-acclaimed drawbacks of naphthoquinones, while still affecting tumour cell viability through hitherto unknown modes of action.

Herein, supported by a unique machine intelligence platform and without resorting to NP tagging, we disclose that the hydroquinone form of **1** is a potent and reversible modulator of 5-lipoxygenase (5-LO). Moreover, hydroquinone **1** binds to an allosteric pocket with a unique molecular recognition pattern. Crucially, we establish an unprecedented link between 5-LO inhibition by **1** and its well-documented anticancer activity.

## Results and discussion

As part of our NP target identification program, we set out to investigate the pharmacology of **1**. We employed a machine learning method (SPiDER)^12^,^13^ that uses a consensus of self-organizing maps built from physicochemical and pharmacophore features. Tessellation of the chemical feature space provides an intuitive means for data visualization and prediction of potential drug target counterparts. In summary, co-clustering of **1** with any given reference ligands suggests a similar ligand–target relationship. Additionally, the method calculates a *p*-like value to rank order qualitative predictions, *i.e.* a binary bind/do not bind output. The approach had been validated with NPs, to which new biology was inferred.^5^,^7^,^13^,^14^ In contrast to other tools using structural fingerprints,^15^,^16^ several targets were confidently predicted for **1** with SPiDER^12^ (*cf.* ESI†). From an algorithm standpoint, competing tools arguably underperformed in this case due to the employed descriptors. In fact, **1** presents a scaffold scarcely explored in target-based screens, which impacts directly in tools using substructural fingerprints, but not as prominently in SPiDER.

With confident SPiDER predictions in hand, we prioritized 228 kinases, 5-LO, and selected G-protein coupled receptors (GPCRs) and transient receptor potential channels for additional analyses, since they had also been confidently predicted for lapachol – an isomer of **1** (*cf.* ESI†) – and downstream assays were readily accessible. We sought additional prediction confidence by building the Drug–Target Relationship Predictor (DEcRyPT) machine intelligence workflow that uses regression random forest technology as an orthogonal learning approach to self-organizing maps. In doing so, we aimed at curating confident predictions from SPiDER by estimating an affinity value for **1** against the 236 pre-selected targets. DEcRyPT was built using manually and automatically curated ChEMBL22 data to solely include relevant bioactivity information for model assembly. The bioactivity annotations were normalized by transforming the affinity data into a –log_10_ value (*p*_Affinity_). The CATS2 topological pharmacophore descriptors were then calculated for each reference ligand (MOE, CCG Canada implementation).^17^ In short, CATS2 combinatorially autocorrelates pharmacophore features within a molecule (positive/negative charge, lipophilic, aromatic, hydrogen bond donor/acceptor) up to a topological distance of 10 bonds. Given the ‘fuzziness’ of the molecular descriptor, the resulting high dimensional vector can be used to leverage machine intelligence in the absence of apparent ligand structure similarity, which is arguably ideal for both *de novo* designed compounds^12^ and NPs.^18^ The built models were subjected to stratified 10-fold cross-validation to assess quality. An average mean absolute error of 0.533 ± 0.103 log units suggests the general utility of the computed models. Moreover, the models support a range of different activities for **1**, providing complementary information to clustering algorithms, such as SPiDER, and further allowing screening prioritizations.

**Fig. 2:**
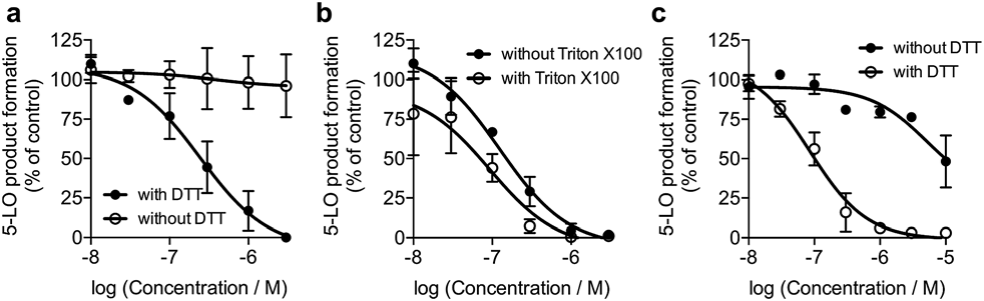
β-Lapachone, **1**, inhibits human 5-lipoxygenase (5-LO). (a) Inhibition of 5-LO in a cell-free assay in presence or absence of 1 mM dithiothreitol (DTT). IC_50_ (with DTT) = 0.24 μM ± 0.13 log units, *n* = 3. IC_50_ (without DTT) > 30 μM, *n* = 3. Control: zileuton, IC_50_ = 1 μM. (b) Inhibition of 5-LO by **1** plus 1 mM DTT in presence or absence of 0.01% Triton X100. IC_50_ (with Triton X100) = 0.09 μM ± 0.21 log units, *n* = 3; IC_50_ (without Triton X100) = 0.12 μM ± 0.14 log units, *n* = 3. (c) Inhibition of 5-LO activity in neutrophil homogenates in presence or absence of 1 mM DTT. IC_50_ (with DTT) = 0.09 μM ± 0.13 log units, *n* = 3; IC_50_ (without DTT) = 5.2 μM ± 0.46 log units, *n* = 3. Inhibition by zileuton is independent of DTT.

We screened **1** against 45 kinases, 4 GPCRs (EP_1–4_), 2 transient receptor potential channels (TRPV1 and TRPM8), and 1 enzyme (5-LO) using functional assays. At a single concentration (150 μM; *cf.* ESI†), we found that **1** interfered with the kinase assay technology and presented only weak TRP channel effects. Also in agreement with DEcRyPT, **1** presented potent activity against EP_3_ and 5-LO, especially in the latter case. Crucially, the observed range of activities against unrelated proteins suggests specific target recognition (*cf.* ESI†). In fact, **1** displayed concentration-dependent effects against 5-LO and ligand efficiency >0.30, indicating that potent entities inspired by its scaffold may be obtained *via* medicinal chemistry. Compound **1** potently inhibited 5-LO in a cell-free functional assay and in reducing conditions (IC_50_ = 0.24 μM ± 0.13 log units; Fig. 2a; Table 1). An orthogonal 5-LO functional assay corroborated the obtained data (*cf.* ESI†). Albeit more modest, **1** inhibited EP_3_ with potency identical to that predicted by DEcRyPT. Our data thus show the accuracy and general utility of our method for the prediction of target affinities. Significantly, no aggregation of **1,** which could lead to artefactual readouts, was measured through dynamic light scattering at relevant concentrations (*cf.* ESI†). The presence or absence of 0.01% Triton X100 also led to identical concentration-response curves in cell-free 5-LO inhibition assays (Fig. 2b), which again rules out unspecific interference by **1**. We then analysed 5-LO inhibition in intact human neutrophils stimulated with A23187 and exogenous arachidonic acid to assess the efficiency of **1** as a leukotriene biosynthesis inhibitor.^19^ β-Lapachone suppressed 5-LO product formation with an IC_50_ value of 8.6 μM ± 0.10 log units (*cf.* ESI†). The reduced potency of **1** in inhibiting 5-LO product formation in neutrophils could be due to hampered cellular uptake of the compound or competition with endogenous factors. Still, when supplementing neutrophils with 1 mM dithiothreitol, **1** inhibited 5-LO with an IC_50_ value of 0.42 μM ± 0.10 log units (*cf.* ESI†), which is in accordance with data from the cell-free assay. Thus, **1** is likely not fully reduced in the native neutrophil environment. Moreover, **1** potently inhibited 5-LO in neutrophil homogenates with 1 mM dithiothreitol with an IC_50_ value of 85.5 nM ± 0.13 log units (Fig. 2c), in line with the cell-free 5-LO assay.

**Table 1:** Affinity predictions with DEcRyPT for β-lapachone

Target name	Prediction^*a*^	MAE^*b*^	Experimental^*c*^
Kinases (*n* = 228)	<4.0–6.4	0.268–0.925	n.d.
5-LO	4.5	0.427	6.6^*d*^, 5.6^*e*^
TRPV1	<4.0	0.556	∼4.0
TRPM8	<4.0	0.465	<4.0
EP_1_	<4.0	0.745	<4.0
EP_2_	5.3	0.487	∼4.0
EP_3_	5.1	0.507	4.7
EP_4_	4.4	0.591	∼4.0
PDE5	<4.0	0.642	<5.0

^*a*^Prediction = *p*_Affinity_ – variance; where *p*_Affiinty_ –log(IC/EC_50_, *K*_D/i_).

^*b*^Mean absolute error.

^*c*^–log(IC_50_ or *K*_D_).

^*d*^Metabolite detection assay.

^*e*^Indirect fluorogenic readout. n.d. = not determined due to assay interference.

**Fig. 3:**
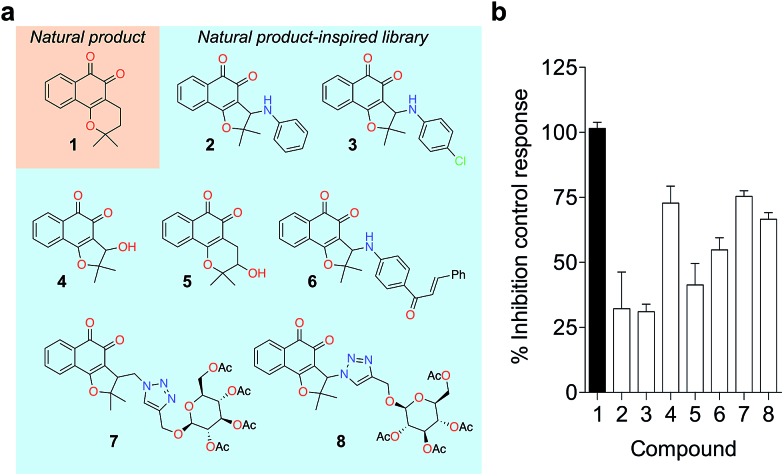
Focused library of β-lapachone-inspired entities. (a) Structures of the synthesized analogues **2–8**. (b) Relative inhibitory activities against 5-LO (cell-free assay, Cerep, France) at 5 μM, *n* = 2. Bars depict the range.

To probe for metalloenzyme selectivity, we screened **1** against 12-LO and 15-LO using two different methods. Weak inhibition (IC_50_ > 30 μM) was obtained in both cases, which highlights selectivity of **1** for 5-LO (*cf.* ESI†). Screening against the solvent-exposed Zn^2+^-containing phosphodiesterase 5 (PDE5) also revealed inactivity, suggesting that **1** is not a general metal chelator (Table 1). Overall, we provide robust evidence that **1** is a true 5-LO inhibitor. Notably, the scaffold of **1** is not exploited among 5-LO ligands despite preserving the pharmacophore features for target engagement (*cf.* ESI†). Thus, similarity searches with commonly used fingerprints would likely fail in identifying the **1**–5-LO relationship despite the existence of other *ortho*-quinone 5-LO inhibitors, *i.e.* the ligand–target association is non-obvious. To further probe the specific 5-LO recognition we expanded our hit by synthesizing a focused library of racemic β-lapachone-inspired entities (Fig. 3a). Generally, the *in situ* bromination of an appropriate starting material afforded the respective key intermediates, which were subsequently functionalized with the required nucleophilic species.^20^–^22^ A range of inhibition potencies were obtained for racemic mixtures of compounds **2–8** in cell-free 5-LO assays, supporting the importance of the substitution pattern for bioactivity and the specific, directed interactions of **1** with 5-LO (Fig. 3b).

To understand the molecular basis of 5-LO inhibition by **1** we conducted the cell-free functional assay in the absence of dithiothreitol, which led to negligible 5-LO modulation up to a concentration of 30 μM (Fig. 2a). From a learning algorithm point of view the result is puzzling as the drug target predictions were carried out with *ortho*-quinone **1**. However, it is possible that the active species are ill-annotated for a fraction of quinone/*ortho*-quinone 5-LO inhibitors included in ChEMBL22 and that were used as training dataset; this can justify the prediction outcome. Nonetheless, our machine intelligence is able to confidently predict hydroquinone **1** as a 5-LO modulator. In a broader perspective, the obtained result underscores the need to better profile quinones whenever found active in functional assays. The enzymatic result was also reproducible with homogenized human neutrophils, *i.e.* without dithiothreitol the inhibitory potency of **1** is sharply reduced (IC_50_ = 0.085 μM *vs.* 5.2 μM, Fig. 2c). Hence, our data not only shows that hydroquinone **1** is the active species against 5-LO, but also reinforces there is insufficient hydroquinone formation within neutrophils. However, from an anticancer activity standpoint, the marked reducing environment within cancer cells and microenvironment, *e.g.* through glutathione or NQO1, may present the ideal setting for phenotype modulation by **1** in cells with 5-LO overexpression. Next, we confirmed that activity of 5-LO could be reinstated upon wash-out of **1**, and that a variation of arachidonic acid concentration (2.5–40 μM) led only to minor alterations of the potency of **1** (*cf.* ESI†). Taken together, our data robustly advocates for a reversible and allosteric modulation of 5-LO by hydroquinone **1**, as a potential means for mediating the anticancer activity.^23^ This result not only agrees with the absence of modulation of related (12-/15-LO) and unrelated (PDE5) metal-containing targets, but also contrasts with a report suggesting chelation of the Fe^3+^ centre in IDO1.^24^

**Fig. 4:**
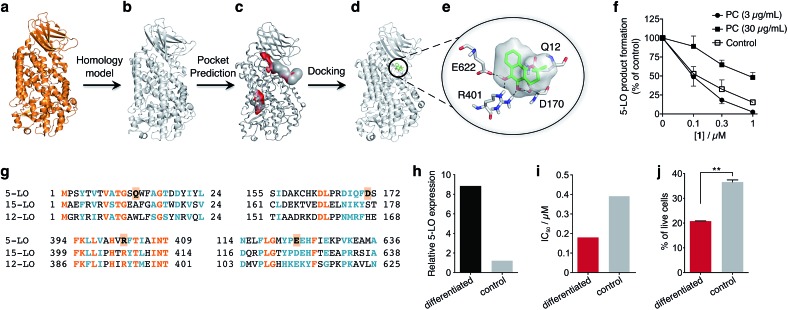
Binding model of hydroquinone **1** to 5-lipoxygenase (5-LO) and relationship with the anticancer activity. (a) Mutant 5-LO used as template (PDB ; 3V98). (b) Homology model of wild-type human 5-LO. (c) Predicted binding pockets with volume > 110 Å^3^. (d) Docking pose of hydroquinone **1** into the predicted allosteric pocket. (e) Detail of the predicted interactions between **1** and 5-LO. (f) Competition assay (IC_50_ curve) between **1** and phosphatidylcholine (PC), *n* = 3. IC_50_ (3 μg mL^–1^, PC) = 100 nM; IC_50_ (30 μg mL^–1^, PC) = 1000 nM. Data advocates for a competition event. (g) Sequence alignment between 5-, 12- and 15-LO. Orange: full match; blue: partial match. Residue counterparts for hydroquinone **1** are highlighted in orange boxes. (h) 5-LO protein expression. Control: HL-60 cells; differentiated: DMSO-stimulated HL-60 cells. (i) IC_50_ values for **1** against both HL-60 cell line groups. IC_50_ (differentiated) = 0.18 μM; IC_50_ (control) = 0.39 μM, *n* = 1. (j) Percentage of live HL-60 cells in the differentiated and control groups when treated with 0.5 μM of **1**, *n* = 3. Statistics: two-tailed *t*-Student test; **p < 0.005.

**Fig. 5:**
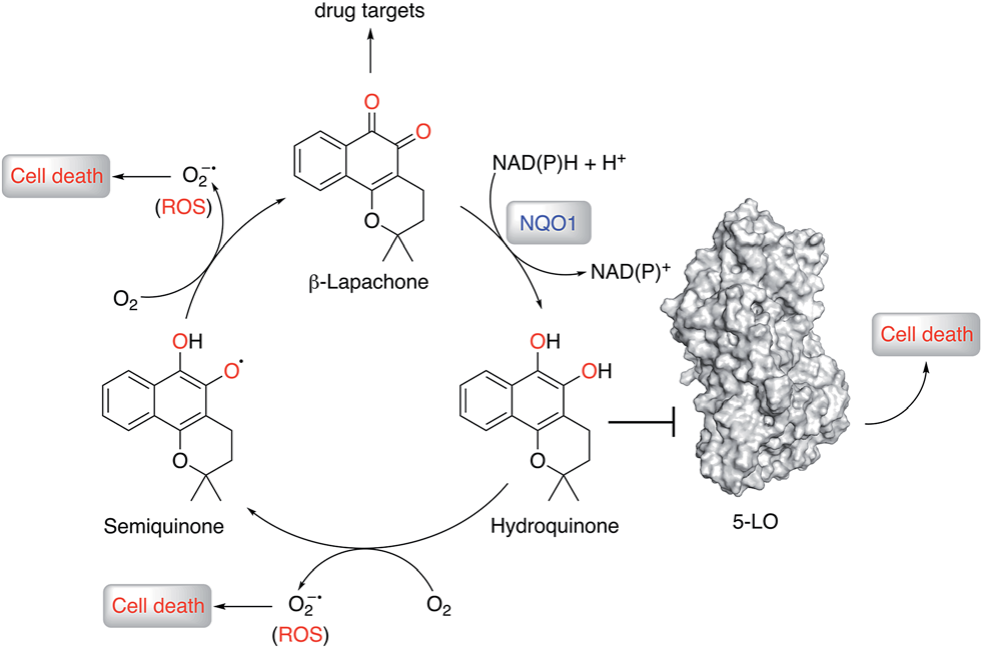
Proposed mechanism of anticancer activity of β-lapachone. β-Lapachone is reduced *in situ* by NQO1 (or glutathione) to the corresponding hydroquinone form, which inhibits 5-lipoxygenase (5-LO) and elicits cancer cell death. β-Lapachone and its hydroquinone form may present additional drug targets and form reactive oxygen species (ROS) that, together with 5-LO, contribute to the overall phenotypic effects.

To obtain insights into the putative binding mode of hydroquinone **1**, we built a homology model for the wild type 5-LO using the apo mutant structure as template (Fig. 4a and b). We then predicted binding pockets with volume > 110 Å^3^, given that only a small fraction of pockets with smaller volumes accommodate ligands (Fig. 4c).^25^ Several 5-LO ligands have been predicted to sit in an allosteric site at the C2-like and catalytic domains' interface^25^ – a region well known to accommodate phospholipids and being critical for 5-LO function and dynamics.^26^,^27^ Supported by our data and literature, we docked hydroquinone **1** with GOLD^28^ into this predicted site (Fig. 4d). The resulting model suggests that hydroquinone **1** binds through hydrogen bonds to D170, R401 and E622, and performs an amide–π stacking with Q12 (Fig. 4e), at the C2-like and catalytic domains' interface. Disruption of the C2–catalytic domain interaction is known to increase 5-LO activity,^29^ which can be counteracted by hydroquinone **1**. Moreover, molecular docking of a select enantiomeric pair suggests that molecular recognition is sensitive to stereogenic centre configuration and that future study of enantiopure entities may provide 5-LO-tailored inhibitors (*cf.* ESI†). We next performed competition assays between **1** and phosphatidylcholine, which binds to the predicted groove. Increasing concentrations of phosphatidylcholine promote the 5-LO product formation (*cf.* ESI†) and markedly diminish the 5-LO blocking efficiency of hydroquinone **1** (Fig. 4f), providing evidence for competitive binding. Furthermore, the alignment of 5-LO, 12-LO and 15-LO (Fig. 4g) sequences shows dissimilarities in the predicted binding pocket region, which may partly explain the weak effects of **1** against the latter two. Altogether, the excellent agreement between the *in silico* models and biochemical data suggests a direct interaction between hydroquinone **1** and 5-LO, through a hitherto unknown allosteric recognition mechanism at the C2–catalytic domains' interface. Finally, to ascertain the importance of allosteric binding and 5-LO inhibition for the anticancer activity of **1** we conducted cell-viability assays with the HL-60 cell line. This leukemia cell line does not overexpress 5-LO except when differentiated (Fig. 4h and ESI†).^30^,^31^ Treatment of both groups with **1** showed that cells overexpressing 5-LO were more sensitive than the control (Fig. 4i and ESI†), displaying IC_50_ values of 0.18 μM and 0.39 μM, respectively. Indeed, the result is statistically significant (Fig. 4j), which endorses 5-LO as an anticancer target for **1***in vitro* (Fig. 5). The result ultimately confirms our hypothesis that in cancer cells the strongly reducing environment efficiently generates hydroquinone **1** for 5-LO modulation.

## Conclusions

Herein, we provide innovative machine intelligence to deconvolute targets of phenotypic hits and accurately predict affinities. However, DEcRyPT presents caveats; it depends on several high-quality positive/negative data points, spanning a wide range of activities for model building. These requirements are not met for ChEMBL targets that were discarded from DEcRyPT. Still, interpreting the output of an ensemble of decision trees is intuitive for chemists and biologists, to whom such models may be useful. Moreover, random forests do not require data pre-processing, *e.g.* scaling descriptors. These are considerable advantages compared to other algorithms, including deep neural networks. The results also suggest that sparse topological pharmacophores efficiently encode target relationships between similar ligands.

Despite numerous reports and studies on the biology of **1** and high interest in 5-LO over the past two decades, no association between them had been established by expert researchers. The machine intelligence platform described here was key to unravel this unprecedented link. We thoroughly validate allosteric inhibition of 5-LO by hydroquinone **1** and disclose its significance in a cancer cell line. Thus, further studies on the compound–target–disease relationship are warranted. These studies might be daunting, considering the challenges of developing naphthoquinones as therapeutics; one must consider that **1** perturbs multiple targets, some of which remain unknown. Nonetheless, we provide proof-of-concept that smart computational technologies can effectively advance early natural product-based drug discovery, be a swift alternative to chemoproteomics and work as a general strategy for systems pharmacology studies.

## Conflicts of interest

T. R. and G. J. L. B. are inventors on a pending patent related to technology described in this work.

## Supplementary Material

Supplementary information
