# Supplementary material for: Machine intelligence decrypts β-lapachone as an allosteric 5-lipoxygenase inhibitor
Source: Chem Sci. 2018 Jul 17;9(34):6899–903. doi: 10.1039/c8sc02634c (PMC6138237; doi:10.1039/c8sc02634c)
Supplement: Supplementary file 1 [file SC-009-C8SC02634C-s001.pdf]

Electronic Supplementary Material (ESI) for Chemical Science.
This journal is © The Royal Society of Chemistry 2018

Electronic Supplementary Information

Machine intelligence decrypts β-lapachone as an allosteric 5-lipoxygenase inhibitor

Correspondence should be addressed to T.R. and G.J.L.B.:
T.R.: tiago.rodrigues@medicina.ulisboa.pt;
G.J.L.B.: gbernardes@medicina.ulisboa.pt, gb453@cam.ac.uk

Table of contents

1. Experimental section S3
1.1 Computational studies S3
1.1.1 Database analyses S3
1.1.2 Target prediction S3
1.1.3 Binding pocket prediction S3
1.1.4 Molecular docking S4
1.2 Chemistry S4
1.2.1 General considerations S4
1.2.2 Synthesis of β-lapachone, lapachol and analogues S4
1.3 Biophysical methods S9
1.3.1 Dynamic light scattering S9
1.3.2 Intrinsic tryptophan fluorescence S9
1.4 Biology S9
1.4.1 Kinase assays S9
1.4.2 GPCR assays S11
1.4.3 TRP assays S11
1.4.4 Lipoxygenase assays S12
1.4.5 Phosphodiesterase 5 assay S13
1.4.6 Minimum inhibitory concentration (MIC) assays S14
1.4.7 Cancer cell assays S14
2. Supplementary data and discussion S15
2.1 Analyses of compound databases S15
2.2 Target prediction S16
2.3 Kinase, ion channel and enzyme screening S18
2.4 β-Lapachone-inspired chemical library S23
2.5 Anti-microbial screening S23
2.6 Mechanism of 5-LO inhibition by β-lapachone S24
2.7 Anticancer assays S26
2.8 Spectral data S28
2.9 Dynamic light scattering S38
3. References S39

1 Experimental section

1.1 Computational studies

1.1.1 Database analyses

A natural product collection comprising 515,581 structures from the Universal Natural Product Database, ZINC database and Traditional Chinese Medicines database was assembled. The library was standardized with the "wash" function in MOE 2015.10 (Chemical Computing Group, Canada) and duplicates were filtered out to afford 428,308 unique natural products. Fragment-like entities in ZINC15 and FDA-approved drugs were collected from ZINC15 and DrugBank v5.0, respectively, and processed identically. PAINS and REOS filters were employed as implemented in Canvas (Schrödinger LLC) to afford 342,114 fragment-like entities from ZINC15, 36,365 fragment-like PAINS/REOS-free natural products, and 37.942 fragment-like PAINS/REOS-containing natural products. Murcko scaffolds, Morgan fingerprints (radius 2, 2048 bits), RDKit and CATS descriptors were calculated in KNIME through RDKit and MOE native nodes. Principal component analyses were performed in KNIME. Drug-likeness was computed with DataWarrior.[1] Data was plotted with Python 2.7.10.

1.1.2 Target prediction

SPiDER. Target prediction was carried out on the publically available web server (www.cadd.ethz.ch/software/spider.html) as previously reported.[2-6] In short, β-lapachone and lapachol were projected onto self-organizing maps together with reference compounds from the COBRA database.[7] Chemical structures are processed with the "wash" function of the Molecular Operating Environment (MOE, Chemical Computing Group, Montreal, Canada), prior to description with the CATS2[8] and MOE2D descriptors. Predictions are carried out by calculating the Euclidean distances of the molecules to the reference compounds in COBRA. The output comprises target families at a confidence level of $p <$ 5%. The distances are converted to p values, according to a pre-calculated background distribution of distances between molecules annotated to bind different targets. The arithmetic average of these p values serves as confidence score for the target prediction. With the background distribution of confidence scores, each prediction can be associated with another p value that indicates the statistical significance of the prediction.[2] SEA[9] and SuperPred[10] predictions were performed from the respective web servers.

DEcRyPT. Ligand and bioactivity data for targets of interest was collected from ChEMBL22. Ligands were normalized with the "wash" function in MOE 2015.10 and bioaffinity data (K_i, K_D, IC_{50} or EC_{50}) transformed to the respective antilog value (p*Affinity*). Regression random forest models were built for each individual target using CATS2 descriptors. For each target, 500 models were built without tree depth and a minimum split node size of 2. The models were subjected to 10-fold cross validation and mean average errors calculated.

1.1.3 Binding pocket prediction

A homology model of 5-lipoxygenase (5-LO) was constructed with Swiss-Model (https://swissmodel.expasy.org), by reinstating the native enzyme sequence into a mutant *apo* (PDB 3V98[11]) 5-LO structure. Hydrogen atoms, charges and energy minimization (Amber10:EHT force field) was performed with MOE 2015.10 (Chemical Computing Group,

Montreal, Canada), as well as binding pocket prediction. Only pockets with a volume >110 Å^3 were considered for further study.[12] The stereochemical quality of the model was assessed through Ramachandran plots.

1.1.4 Molecular docking

The structure of β-lapachone was "washed" and energy minimized with MOE2015.10, prior to docking into the predicted binding pockets with GOLD 5.4.1.

Docking runs were performed with the *apo* 5-LO model. Default settings, including the scoring function ChemPLP were used.[13] Several docking runs using different pocket centres were performed. Five hundred genetic algorithm runs were performed, and the top 10% poses were saved for manual inspection.

1.2 Chemistry

1.2.1 General considerations

Building blocks and solvents were purchased from ABCR Chemicals, Sigma Aldrich, Alfa Aesar, Acros, Fluka or TCI Deutschland and used without further purification. Proton and carbon nuclear magnetic resonance (^{1}H and ^{13}C NMR) spectra were recorded on a Bruker AVANCE DRX400 MHz and Varian Mercury 500 MHz spectrometers. All chemical shifts are quoted on the δ scale in ppm using with TMS as an internal reference. Coupling constants (*J*) are reported in Hz with the following splitting abbreviations: s = singlet, d = doublet, t = triplet, dd = doublet of doublets, td = triplet of doublets, m = multiplet. All compounds present ≥ 95% purity unless otherwise stated.

1.2.2 Synthesis of lapachol, β-lapachone and analogues

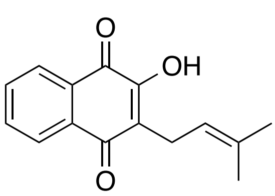

Lapachol. Lapachol (2-hydroxy-3-(3'-methyl-2'-butenyl)-1,4-naphthoquinone) was extracted from the heartwood of *Tabebuia* sp. (Tecoma). Initially, a saturated aqueous sodium carbonate solution was prepared and added to the sawdust of ipe tree. Upon observing rapid formation of lapachol sodium salt (deep red coloration), hydrochloric acid was added, allowing the precipitation of lapachol. The solution was then filtered and a yellow solid was obtained. This solid was purified by a series of recrystallizations with hexane as a solvent. Lapachol was then obtained as crystalline yellow solid with high purity (>99%). ^{1}H NMR (400 MHz, $CDCl_3$, 303 K) δ: 8.11 (dd, 1H, *J* = 7.6 and 1.4 Hz), 8.06 (dd, 1H, *J* = 7.6 and 1.4 Hz), 7.74 (td, 1H, *J* = 7.6 and 1.4 Hz), 7.66 (td, 1H, *J* = 7.6 and 1.4 Hz), 7.36 (s, 1H), 5.25-5.17 (m, 1H), 3.31 (d, 2H, *J* = 7.3 Hz), 1.79 (s, 3H), 1.69 (s, 3H). ^{13}C NMR (100 MHz, $CDCl_3$, 303 K) δ: 184.4, 181.5, 152.5, 134.7, 133.7, 132.7, 129.3, 126.6, 125.9, 123.3, 119.5, 25.6, 22.5, 17.8.

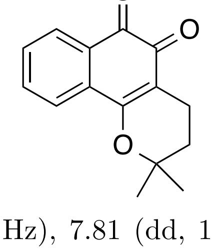

β-Lapachone. Sulfuric acid was slowly added to lapachol (1 mmol, 242 mg) until complete dissolution of the quinone and formation of a red solution. The solution was then poured into ice and the precipitate formed was filtered off and washed with water. β-Lapachone was recrystallized in ethanol and obtained as an orange solid (240 mg, 99% yield); mp 153-155 °C. ^{1}H NMR (400 MHz, $CDCl_3$, 303 K) *δ*: 8.06 (dd, 1H, *J* = 7.6 and 1.4 Hz), 7.81 (dd, 1H, *J* = 7.8 and 1.1 Hz), 7.65 (ddd, 1H, *J* = 7.8, 7.6 and 1.4 Hz), 7.51 (td,

1H, J = 7.6, 7.6 and 1.1 Hz), 2.57 (t, 2H, J = 6.7 Hz), 1.86 (t, 2H, J = 6.7 Hz), 1.47 (s, 6H). ^{13}C NMR (100 MHz, $CDCl_3$, 303 K) δ: 179.8, 178.5, 162.0, 134.7, 132.6, 130.6, 130.1, 128.5, 124.0, 112.7, 79.3, 31.6, 26.8, 16.2. Data are consistent with those reported in the literature [14, 15].

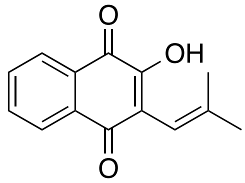

Nor-Lapachol. Nor-lapachol was synthesized by the Hooker oxidation[16] and data are consistent with those reported in the literature.[17-19] In a 500 mL flask was added 4.84 g of lapachol and then 40 mL of THF. Separately, a solution of 2.4 g of anhydrous Na_2CO_3 in 50 mL of H_2O was prepared, which was also added to the flask, forming a dark red solution. At 60 °C, 6 mL of 30% H_2O_2 were added (10 drops at a time) over 1 hour, until a pinkish solution was formed. The solution was cooled (0 °C to -2 °C) and concentrated HCl was then added dropwise under stirring until a white precipitate appeared. The mixture was left in the refrigerator for 2 hours and then filtered to obtain a white solid (70% yield). This substance was then dissolved in 32 mL of THF and a solution of 1.4 g of Na_2CO_3 in 59 mL of H_2O was added. 20 mL of 25% NaOH, followed by 12 g of $CuSO_4.5H_2O$, in 59 ml of H_2O were added under constant stirring. The solution was left in a water bath for 1 hour and 45 minutes, then filtered on Celite® (infusion ground) to give a deep red mother liquor. The solution was acidified with concentrated HCl to form an orange precipitate, which was filtered and washed successively with distilled H_2O until complete neutralization. Nor-lapachol was obtained as an orange solid (160 mg, 0.7 mmol, 70% yield); mp 121-122 °C.[16] ^{1}H NMR (400 MHz, $CDCl_3$, 303 K) δ: 8.13 (ddd, 1H, J = 7.5, 1.5 and 0.5 Hz), 8.10 (ddd, 1H, J = 7.5, 1.5 and 0.5 Hz), 7.76 (td, 1H, J = 7.5, 7.5 and 1.5 Hz), 7.69 (td, 1H, J = 7.5, 7.5 and 1.5 Hz), 6.03-5.99 (m, 1H), 2.0 (d, 3H, J = 1.5 Hz), 1.68 (d, 3H, J = 1.2 Hz). ^{13}C NMR (100 MHz, $CDCl_3$, 303 K) δ: 184.7, 181.5, 151.1, 143.6, 134.9, 133.0, 132.9, 129.5, 126.9, 126.0, 120.9, 113.6, 26.5, 21.7.

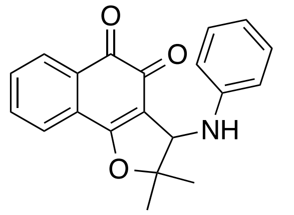

3-Arylamino-nor-β-lapachone. Nor-lapachol (228 mg, 1.0 mmol) was dissolved in 25 mL of dichloromethane, followed by the addition of 2 mL of bromine. A bromo intermediate precipitated immediately as an orange solid. Dicholoromethane was added and the solvent evaporated in vacuum to remove bromine. Aniline (2 mmol) was added and the mixture was stirred overnight. The crude reaction mixture was poured into 50 mL of water. The organic phase was separated and washed with 10% HCl (3 × 50 mL), dried over sodium sulfate, filtered, and evaporated under reduced pressure to yield a solid, which was purified by column chromatography in silica gel and eluted with an increasing polarity gradient mixture of hexane and ethyl acetate (9/1 to 7/3).[20, 21] 3-Arylamino-nor-β-lapachone was obtained as a red solid (303 mg, 95% yield); mp 126-128 °C. ^{1}H NMR (400 MHz, $CDCl_3$, 303 K) δ: 8.00 (d, 1H, J = 7.4 Hz), 7.62 (dt, 1H, J = 14.8 and 7.4 Hz), 7.54 (dd, 1H, J = 10.5 and 4.2 Hz), 7.10 (t, 1H, J = 7.8 Hz), 6.66 (t, 1H, J = 7.3 Hz), 6.50 (d, 1H, J = 7.9 Hz), 4.72 (s, 1H), 3.87 (sl, 1H), 1.59 (s, 3H), 1.50 (s, 3H). ^{13}C NMR (100 MHz, $CDCl_3$, 303 K) δ: 181.17, 175.62, 169.77, 147.58, 134.84, 132.74, 131.42, 129.74, 129.56, 127.69, 125.29, 118.35, 115.47, 113.36, 97.09, 61.92, 27.52, 22.00. Data are consistent with those reported in the literature.[22]

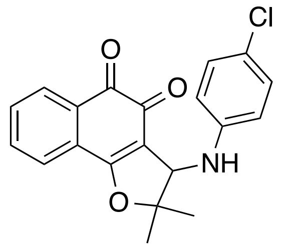

p-Chloro-3-arylamino-nor-β-lapachone. p-Chloro-3-arylamino-nor-β-lapachone synthesized as aforementioned and obtained as a red solid (317 mg, 0.9 mmol, 90% yield); mp 210-214 °C). ^{1}H NMR (400 MHz, $CDCl_3$) δ: 8.1 (ddd, 1H, J = 7.9, 2.2 and 0.7 Hz), 7.72-7.60 (m, 3H), 7.13 (dd, 2H, J = 6.7 and 2.1 Hz), 6.5 (dd, 2H, J = 6.7 and 2.1 Hz), 4.75 (d, 1H, J = 5.7 Hz), 1.66 (s, 3H), 1.56 (s, 3H); ^{13}C NMR (100 MHz, $CDCl_3$) δ: 180.8, 175.3, 169.6, 145.8, 134.6, 132.6, 131.1, 129.5, 129.1, 127.2, 125.0, 122.6, 114.6, 114.1, 96.6, 61.7, 27.3, 21.7.

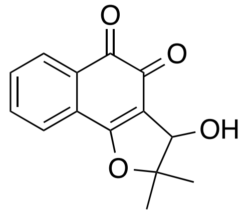

3-Hydroxy-nor-β-lapachone. A solution of nor-lapachol (228 mg, 1.0 mmol) in 25 mL of dichloromethane, and 2 mL of bromine was prepared. The bromo intermediate precipitated immediately as an orange solid. The reaction mixture was transferred to the separatory funnel and extracted with sodium bisulfite (3 × 10 mL) to form the hydroxylated product. The product was obtained as an orange solid (170 mg, 70% yield); mp 110-112 °C. ^{1}H NMR (400 MHz, $CDCl_3$, 303 K) δ: 7.96 (dd, 1H, J = 7.5 and 0.7 Hz), 7.58 (dtd, 2H, J = 8.8, 7.5 and 1.3 Hz), 7.51 (td, 1H, J = 7.4 and 1.6 Hz); 4.95 (s, 1H), 3.82 (s, 1H), 1.56 (s, 3H), 1.40 (s, 3H). ^{13}C NMR (100 MHz, $CDCl_3$, 303 K) δ: 181.5, 176.4, 171.2, 134.8, 132.8, 131.4, 129.6, 127.7, 125.4, 117.8, 97.2, 75.3, 26.8, 20.8. Data are consistent with those reported in the literature.[23]

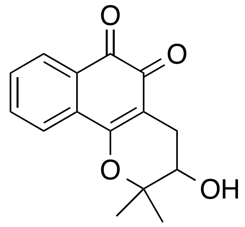

3-Hydroxy-β-lapachone: To a solution of lapachol (100 mg, 0.42 mmol) in dichloromethane (20 mL) was added m-chloroperbenzoic acid (90 mg, 0.52 mmol) and the mixture was stirred at room temperature for 24 h. The reaction mixture was washed with a saturated solution of sodium bicarbonate, water, dried over magnesium sulfate, and the solvent was evaporated. The residue was purified by column chromatography on silica gel using chloroform/ethyl acetate (2:1) as eluent to afford the product as a red solid (54 mg, 50% yield); mp 202-204 °C. ^{1}H NMR (400 MHz, $CDCl_3$, 303 K) δ: 7.98 (dd, 1H, J = 7.6 and 0.9 Hz), 7.74 (d, 1H, J = 7.8 Hz), 7.60 (td, 1H, J = 7.7 and 1.3 Hz), 7.46 (td, 1H, J = 7.6 and 1.0 Hz), 4.18 (dd, 1H, J = 7.3 and 5.4 Hz), 3.14 (dd, 1H, J = 18.1 and 5.4 Hz), 2.91 (dd, 1H, J = 18.1 and 7.4 Hz), 1.56 (s, 3H), 1.53 (s, 3H). ^{13}C NMR (100 MHz, $CDCl_3$, 303 K) δ: 179.3, 178.2, 161.3, 135.2, 131.9, 131.4, 130.3, 129.1, 124.5, 111.3, 81.3, 50.2, 28.2, 26.4, 23.9. Data are consistent with those reported in the literature.[24, 25]

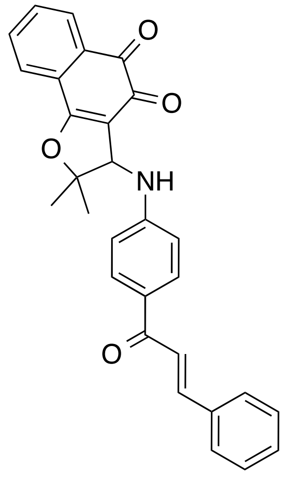

3-((4-Cinnamoylphenyl)amino)-2,2-dimethyl-2,3-dihydronaphtho[1,2-*b*]furan-4,5-dione. The required chalcone was prepared by condensing 4'-aminoacetophenone with the respective aldehyde in the presence of sodium hydroxide in ethanol. An excess of bromine (2 mL) was then added to a cooled solution of nor-lapachol (228 mg, 1 mmol) in 25 mL of dichloromethane. The brominated intermediate was obtained as an orange solid. After the removal of excess bromine, a solution of the respective chalcone (1 mmol) in 25 mL of dichloromethane was added and stirred

overnight. The reaction mixture was concentrated under reduced pressure and the residue was purified by column chromatography in silica gel by eluting with an increasing polarity gradient mixture of hexane and ethyl acetate.[26] The title compound was obtained as a red solid. (157 mg, 0.35 mmol, 35% yield); mp 196-199 °C. ^1H NMR (500 MHz, $CDCl_3$) δ: 7.98 (d, 1H, J = 7.4 Hz), 7.88 (d, 2H, J = 8.4 Hz), 7.69 (dt, 3H, J = 14.8 and 11.6 Hz), 7.59 (t, 3H, J = 7.4 Hz), 7.49 (d, 1H, J = 15.6 Hz), 7.39-7.35 (m, 3H), 6.61 (d, 2H, J = 8.7 Hz), 4.91 (d, 1H, J = 7.3 Hz), 1.69 (s, 3H), 1.57 (s, 3H). ^{13}C-APT NMR (125 MHz, $CDCl_3$) δ: 187.8, 180.7, 175.2, 170.0, 151.3, 142.9, 135.3, 134.6, 132.7, 131.1, 131.0, 129.9, 129.5, 128.8, 128.2, 128.0, 127.2, 125.2, 121.9, 114.5, 112.2, 96.7, 60.8, 27.4, 21.8.

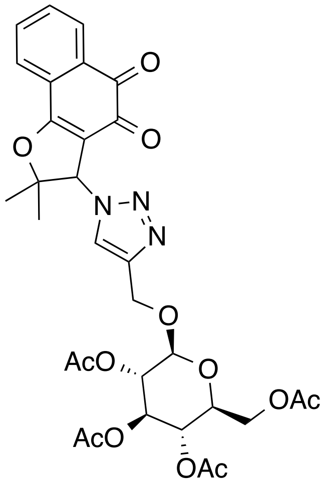

(2*S*,3*S*,4*R*,5*S*,6*S*)-2-(Acetoxymethyl)-6-((1-(2,2-dimethyl-4,5-dioxo-2,3,4,5-tetrahydronaphtho[1,2-*b*]furan-3-yl)-1*H*-1,2,3-triazol-4-yl)methoxy)tetrahydro-2*H*-pyran-3,4,5-triyl triacetate. To a mixture of 3-azido-nor-β-lapachone (269 mg, 1.0 mmol), $CuSO_4 \cdot 5H_2O$ (12.5 mg, 5 mol %) and sodium ascorbate (20 mg, 5 mol%) in 8 mL CH_2Cl_2/H_2O (1:1 v/v), the required alkyne-carbohydrate (1.1 molar equiv.) was added. The mixture was stirred overnight at room temperature. The organic phase was extracted with dichloromethane, dried with $NaSO_4$ and concentrated under reduced pressure. The residue obtained was purified by column chromatography on silica gel using as eluent a gradient mixture of hexane/ethyl acetate with increasing polarity. The nor-β-lapachone-based 1,2,3-triazole-carbohydrate was obtained as a yellow solid (537 mg, 0.82 mmol, 82% yield); mp 88-90 °C. ^1H NMR (400 MHz, $CDCl_3$) δ: 8.20-8.17 (m, 1H), 7.82-7.71 (m, 3H), 7.55/7.51 (s, 1H), 5.98/5.97 (s, 1H), 5.25-5.15 (m, 1H), 5.07 (t, 1H, J = 10 Hz), 5.00-4.78 (m, 3H), 4.67 (t, 1H, J = 7.6 Hz), 4.30-4.10 (m, 2H), 3.77-3.70 (m, 1H), 2.07, 2.01, 2.00, 1.98, 1.97, 1.77 (six singlets observed, 12H), 1.19 (s, 3H), 0.98-0.83 (m, 3H). ^{13}C NMR (100 MHz, $CDCl_3$) δ: 180.0, 180.0, 174.6, 174.5, 171.3, 171.2, 170.7, 170.6, 170.1, 170.1, 169.6, 169.5, 169.4, 144.4, 144.2, 134.8, 134.8, 133.4, 133.4, 131.6, 131.5, 130.0, 130.0, 126.6, 126.6, 125.6, 125.5, 122.6, 122.3, 111.1, 100.2, 99.9, 95.9, 95.8, 72.8, 72.6, 71.9, 71.3, 71.2, 68.3, 68.3, 67.0, 66.9, 63.3, 63.0, 61.8, 61.7, 31.5, 29.0, 27.7, 27.6, 22.6, 22.6, 21.1, 20.7, 20.6, 20.5, 20.5, 20.4, 14.1, 11.4.

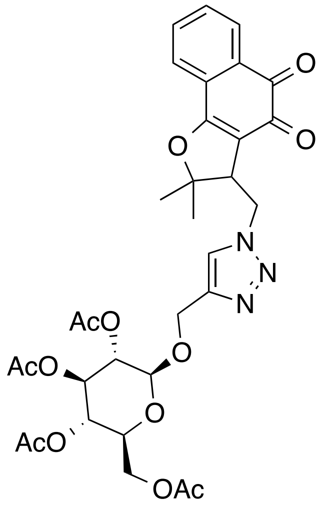

(2*S*,3*S*,4*R*,5*S*,6*S*)-2-(acetoxymethyl)-6-((1-((2,2-dimethyl-4,5-dioxo-2,3,4,5-tetrahydronaphtho[1,2-*b*]furan-3-yl)methyl)-1*H*-1,2,3-triazol-4-yl)methoxy)tetrahydro-2*H*-pyran-3,4,5-triyl triacetate. To a mixture of 256 mg (1 mmol) of the required azide quinone, $CuSO_4 \cdot 5H_2O$ (12.5 mg, 5 mol %) and sodium ascorbate (20 mg, 5 mol%) in 8 mL CH_2Cl_2/H_2O (1:1 v/v), the corresponding alkyne-carbohydrate (1.1 molar equiv.) was added. The mixture was stirred overnight at room temperature. The organic phase was extracted with dichloromethane, dried over $NaSO_4$ and concentrated under reduced pressure. The obtained residue was purified by column chromatography on silica gel using as eluent a gradient mixture of

hexane/ethyl acetate with increasing polarity.[27] The quinone-based 1,2,3-triazole-carbohydrate was obtained as a yellow solid (544 mg, 0.8 mmol, 85% yield); mp 117-119 °C. ^{1}H NMR (400 MHz, $CDCl_3$) δ: 8.06 (bs, 1H), 7.76-7.67 (m, 2H), 7.61-7.59 (bs, 2H), 5.53–5.48 (m, 1H), 5.25-4.70 (m, 8H), 4.25-4.15 (m, 2H), 3.77 (d, 1H, J = 4 Hz), 3.38-3.32 (m, 1H), 2.98-2.93 (m, 1H), 2.08 (s, 3H), 2.04 (s, 3H), 2.01 (s, 3H), 1.97 (s, 3H). ^{13}C NMR (100 MHz, $CDCl_3$) δ: 180.4, 178.7, 175.2, 173.8, 170.6, 170.1, 169.4, 144.7, 134.9, 132.4, 130.5, 129.7, 126.8, 124.6, 123.8, 114.7, 100.0, 84.5, 72.7, 72.0, 71.2, 68.3, 63.2, 61.8, 53.4, 29.7, 20.7, 20.7, 20.6, 20.6.

1.3 Biophysical methods

1.3.1 Dynamic light scattering

Dynamic light scattering (Zetasizer Nano S, Malvern, UK) was used to determine compound colloidal aggregation potential and kinetic solubility. The particle sizes were measured at 25 °C. Water solubility was measured as described elsewhere with successive measurements within 60 minutes.[28] A 100 mM stock solution of β-lapachone was prepared in DMSO, following dilution to deionized water to obtain an analyte solution of 100 µM (0.1% DMSO). Colloidal aggregation was measured through sequential dilutions. Solubility was assessed at 25 µM after 0 and 30 minutes.

1.3.2 Intrinsic tryptophan fluorescence

5-Lipoxygenase (Cat No. ab114310, Abcam) was concentrated to 0.5 µM in buffer (50 mM Tris-HCl, pH 8.0, 5 mM $CaCl_2$). A stock solution of β-lapachone was prepared in DMSO and added to the enzyme solution in a concentration range of 0–10 µM. The final DMSO concentration was kept under 0.1%. Fluorescent measurements were performed using a 1 cm pathlength quartz cuvette. Spectra were collected in an Edinburgh Instruments FLS920 Series Fluorescence Spectrophotometer at 25 °C with fluorescence excitation and scanning emission set to 295 nm and 310 to 450 nm, respectively. All assays were carried out in triplicate.

1.4 Biology

1.4.1 Kinase assays

The ExpresS Diversity Kinase Profile (Ref. P10) was performed at Cerep, SA (Celle l'Evescault, France) on a fee-for-service basis, and as described in Table S1.

Table S1. Kinase assay protocols.

Kinase	Substrate	Measured component	Incubation	Detection
Abl	ATP+Ulight-TK peptide (100 nM)	Phospho-Ulight-TK peptide	60 min / rt	LANCE
EGFR	ATP+Ulight-CAGAGAIETDKE YYTVKD (100 nM)	Phospho-Ulight-CAGAGAIETDKEYYTVKD	15 min / rt	LANCE
AurA	ATP+Ulight-RRRSLLE (100 nM)	Phospho-Ulight-RRRSLLE	15 min / rt	LANCE
IKKα	ATP+Ulight-IkappaBα (100 nM)	Phospho-Ulight-IkappaBα	30 min / rt	LANCE
NEK2	ATP+Ulight-FLGFTYVAP (50 nM)	Phospho-Ulight-FLGFTYVAP	60 min / rt	LANCE
PLK1	ATP+Ulight-FLGFTYVAP (40 nM)	Phospho-Ulight-FLGFTYVAP	60 min / rt	LANCE
c-Met	ATP+Ulight-CAGAGAIETDKEYYTVKD (25 nM)	Phospho-Ulight-CAGAGAIETDKEYYTVKD	60 min / rt	LANCE
EphA2	ATP+Ulight-TK peptide (50 nM)	Phospho-Ulight-TK peptide	30 min / rt	LANCE
EphA3	ATP+Ulight-TK peptide (50 nM)	Phospho-Ulight-TK peptide	60 min / rt	LANCE
EphB4	ATP+Ulight-TK peptide (100 nM)	Phospho-Ulight-TK peptide	90 min / rt	LANCE
FGFR1	ATP+Ulight-CAGAGAIETDKEYYTVKD (100 nM)	Phospho-Ulight-CAGAGAIETDKEYYTVKD	60 min / rt	LANCE
FGFR3	ATP+Ulight-CAGAGAIETDKEYYTVKD (100 nM)	Phospho-Ulight-CAGAGAIETDKEYYTVKD	90 min / rt	LANCE
FGFR2	ATP+Ulight-CAGAGAIETDKEYYTVKD (25 nM)	Phospho-Ulight-CAGAGAIETDKEYYTVKD	15 min / rt	LANCE
IRK	ATP+Ulight-Poly GAT[EAY(1:1:1)]n (50 nM)	Phospho-Ulight-Poly GAT[EAY(1:1:1)]n	10 min / rt	LANCE
VEGFR2	ATP+Ulight-CAGAGAIETDKEYYTVKD (100 nM)	Phospho-Ulight-CAGAGAIETDKEYYTVKD	60 min / rt	LANCE
TRKA	ATP+Ulight-Poly GAT[EAY(1:1:1)]n (5 nM)	Phospho-Ulight-Poly GAT[EAY(1:1:1)]n	10 min / rt	LANCE
JAK3	ATP+Ulight-CAGAGAIETDKEYYTVKD (100 nM)	Phospho-Ulight-CAGAGAIETDKEYYTVKD	60 min / rt	LANCE
Lck	ATP+Ulight-Poly GAT[EAY(1:1:1)]n (25 nM)	Phospho-Ulight-Poly GAT[EAY(1:1:1)]n	30 min / rt	LANCE
Src	ATP+Ulight-Poly GAT[EAY(1:1:1)]n (5 nM)	Phospho-Ulight-Poly GAT[EAY(1:1:1)]n	10 min / rt	LANCE
CDK1	ATP+Ulight-CFFKNIVTPRTPPPSQGK-amide (100 nM)	Phospho-Ulight-CFFKNIVTPRTPPPSQGK-amide	15 min / rt	LANCE
CDK2	ATP+Ulight-CFFKNIVTPRTPPPSQGK-amide (50 nM)	Phospho-Ulight-CFFKNIVTPRTPPPSQGK-amide	30 min / rt	LANCE
ERK2	ATP+Ulight-CFFKNIVTPRTPPPSQGK-amide (100 nM)	Phospho-Ulight-CFFKNIVTPRTPPPSQGK-amide	15 min / rt	LANCE
GSK3β	ATP+Ulight-CFFKNIVTPRTPPPSQGK-amide (100 nM)	Phospho-Ulight-CFFKNIVTPRTPPPSQGK-amide	90 min / rt	LANCE
JNK1	ATP+Ulight-CFFKNIVTPRTPPPSQGK-amide (100 nM)	Phospho-Ulight-CFFKNIVTPRTPPPSQGK-amide	60 min / rt	LANCE
p38α	ATP+Ulight-CFFKNIVTPRTPPPSQGK-amide (100 nM)	Phospho-Ulight-CFFKNIVTPRTPPPSQGK-amide	60 min / rt	LANCE
CaMK2α	ATP+Ulight-CGSGSGRPRTSSFAEG (50 nM)	Phospho-Ulight-CGSGSGRPRTSSFAEG	30 min / rt	LANCE
CHK1	ATP+CREBtide (CKRREILSRRPSYRK) (25 nM)	Phospho-CREBtide (CKRREILSRRPSYRK)	30 min / rt	LANCE
CHK2	ATP+CREBtide (CKRREILSRRPSYRK) (25 nM)	Phospho-CREBtide (CKRREILSRRPSYRK)	15 min / rt	LANCE
MAPKAPK 2	ATP+CREBtide (CKRREILSRRPSYRK) (25 nM)	Phospho-CREBtide (CKRREILSRRPSYRK)	15 min / rt	LANCE
MARK1	ATP+Ulight-RRRSLLE (50 nM)	Phospho-Ulight-RRRSLLE	30 min / rt	LANCE
MNK2	ATP+CREBtide	Phospho-CREBtide	90 min / rt	LANCE

	ATP+CREBtide (CKRREILSRRPSYRK) (25 nM)	Phospho-CREBtide (CKRREILSRRPSYRK)		
Pim2	ATP+CREBtide (CKRREILSRRPSYRK) (25 nM)	Phospho-CREBtide (CKRREILSRRPSYRK)	60 min / rt	LANCE
SIK	ATP+CREBtide (CKRREILSRRPSYRK) (25 nM)	Phospho-CREBtide (CKRREILSRRPSYRK)	90 min / rt	LANCE
Akt1/PKBα	ATP+CREBtide (CKRREILSRRPSYRK) (25 nM)	Phospho-CREBtide (CKRREILSRRPSYRK)	60 min / rt	LANCE
PKA	ATP+Ulight-RRRSLLE (50 nM)	Phospho-Ulight-RRRSLLE	10 min / rt	LANCE
PDK1	ATP+Ulight-FLGFTYVAP (400 nM)	Phospho-Ulight-FLGFTYVAP	90 min / rt	LANCE
PKCβ2	ATP+CREBtide (CKRREILSRRPSYRK) (25 nM)	Phospho-CREBtide (CKRREILSRRPSYRK)	15 min / rt	LANCE
ROCK1	ATP+Ulight-RRRSLLE (50 nM)	Phospho-Ulight-RRRSLLE	30 min / rt	LANCE
SGK1	ATP+Ulight-RRRSLLE (50 nM)	Phospho-Ulight-RRRSLLE	30 min / rt	LANCE
MAP4K4	ATP+Ulight-FLGFTYVAP (50 nM)	Phospho-Ulight-FLGFTYVAP	90 min / rt	LANCE
PAK2	ATP+Ulight-RRRSLLE (50 nM)	Phospho-Ulight-RRRSLLE	60 min / rt	LANCE
PAK4	ATP+Ulight-RRRSLLE (50 nM	Phospho-Ulight-RRRSLLE	60 min / rt	LANCE
TAOK2	ATP+Ulight-FLGFTYVAP (40 nM)	Phospho-Ulight-FLGFTYVAP	60 min / rt	LANCE
IRAK4	ATP+Ulight-FLGFTYVAP (100 nM)	Phospho-Ulight-FLGFTYVAP	90 min / rt	LANCE
RAF-1	ATP+Ulight-ARTKQTARKSTGGKAPRKQLAGCG (50 nM)	Phospho- Ulight-ARTKQTARKSTGGKAPRKQLAGCG (50 nM)	180 min / rt	LANCE

1.4.2 GPCR assays

G-protein coupled receptor (GPCR) assays were performed at Cerep, SA (Celle l'Evescault, France) on a fee-for-service basis. Prostanoid EP1 (Ref G035[29]), EP2 (Ref G036[30]), EP3 (Ref G122[31]), and EP4 (Ref G037[30]) were assayed as described in Table S2.

Table S2. GPCR assay protocols.

GPCR	Assay	Stimulant / ligand	Measured component	Incubation	Detection method
EP_1	Functional agonist	none	Intracellular $[Ca^{2+}]$	rt	Fluorimetry
EP_1	Functional antagonist	PGE_2 (3 nM)	Intracellular $[Ca^{2+}]$	rt	Fluorimetry
EP_2	Functional agonist	none	cAMP	30 min / 37 ºC	HTRF
EP_2	Functional antagonist	PGE_2 (100 nM)	cAMP	30 min / 37 ºC	HTRF
EP_3	Functional agonist	none	Impedance	28 ºC	Cellular dielectric spectroscopy
EP_3	Functional antagonist	Sulprostone (0.1 nM)	Impedance	28 ºC	Cellular dielectric spectroscopy
EP_4	Functional agonist	none	cAMP	10 min / rt	HTRF
EP_4	Functional antagonist	PGE_2 (30 nM)	cAMP	10 min / rt	HTRF

1.4.3 TRP assays

Transient receptor potential channel (TRP) assays were performed at Cerep, SA (Celle l'Evescault, France) on a fee-for-service basis. TRP vanilloid 1 (Ref G106[32]), and TRP melastatin 8 (Ref G0185[33]) were assayed as described in Table S3.

Table S3. TRP channels assay protocols.

TRP	Assay	Stimulant / ligand	Measured component	Incubation	Detection method
V1	Functional agonist	none	Intracellular [Ca^{2+}]	rt	Fluorimetry
V1	Functional antagonist	Capsaicin (30 nM)	Intracellular [Ca^{2+}]	rt	Fluorimetry
M8	Functional agonist	none	Intracellular [Ca^{2+}]	rt	Fluorimetry
M8	Functional antagonist	Icilin (100 nM)	Intracellular [Ca^{2+}]	rt	Fluorimetry

1.4.4 Lipoxygenase assays

Expression, purification and cell-free activity assay of human recombinant 5-LO. *E.coli* (BL21) was transformed with pT3-5-LO plasmid, and recombinant 5-LO protein was expressed at 30 °C as described.[34] Cells were lysed in 50 mM triethanolamine/HCl pH 8.0, 5 mM EDTA, 1 mM phenylmethanesulphonyl fluoride, soybean trypsin inhibitor (60 µg/mL), and lysozyme (1 mg/mL), homogenized by sonication (3 × 15 s), and centrifuged at 40,000 × g for 20 min at 4 °C. The 40,000 × g supernatant (S40) was applied to an ATP-agarose column to partially purify 5-LO as described.[34] Aliquots of semi-purified 5-LO (0.5 µg) were diluted with 1 mL ice-cold PBS containing 1 mM EDTA. Samples were pre-incubated with the test compound or vehicle (0.1% DMSO) with or without Triton X-100 and/or 1 mM DTT, as indicated. After 10 min at 4 °C, samples were pre-warmed for 30 s at 37 °C, and 2 mM $CaCl_2$ plus the indicated concentrations of arachidonic acid (AA) were added to start the formation of 5-LO products. After 10 min, the reaction was stopped by addition of one volume of ice-cold methanol, and the formed 5-LO products were analyzed by RP-HPLC as described.[35] 5-LO products include the all-trans isomers of LTB_4 (tr-LTB_4 isomers) as well as 5(*S*)-hydroperoxy-6-*trans*-8,11,14-*cis*-eicosatetraenoic acid (5-HPETE) and its corresponding alcohol 5(*S*)-hydroxy-6-*trans*-8,11,14-*cis*-eicosatetraenoic acid (5-HETE). For phosphatidylcholine competition assays (3 or 30 µg/ml PC) samples were pre-incubated on ice with β–lapachone with or without 1 mM DDT or vehicle (0.1% DMSO) for 10 minutes. After addition of 2 mM $CaCl_2$ and 20 µM AA the incubation was continued for 10 minutes at 37 °C and after that stopped with methanol.

Neutrophil isolation. Peripheral blood (University Hospital Jena, Germany) was withdrawn from healthy adult volunteers with consent that had not taken any anti-inflammatory drugs during the last 10 days, by venipuncture in heparinized tubes (16 IE heparin/mL blood). The blood was centrifuged at 4000*g* for 20 min at 20 °C for preparation of leukocyte concentrates. Leukocyte concentrates were then subjected to dextran sedimentation and centrifugation on lymphocyte separation medium (LSM 1077, PAA, Colbe, Germany). Contaminating erythrocytes of pelleted neutrophils were removed by hypotonic lysis. Neutrophils were then washed twice in ice-cold PBS and finally resuspended in PBS pH 7.4 containing 1 mg/mL glucose or in PBS pH 7.4 containing 1 mg/mL glucose plus 1 mM $CaCl_2$ (PGC buffer) (purity $>$ 96-97%).

Method A. 5-Lipoxygenase (5-LO), 12-lipoxygenase (12-LO) and 15-lipoxygenase-2 (15-LO-2) inhibition assays were performed at Cerep, SA (Celle l'Evescault, France) on a fee-for-service basis (Ref 0772[36], 0883[37] and 0893[38], respectively), as described in Table S4.

Table S4. Lipoxygenase assay protocols.

Assay	Substrate	Measured component	Incubation	Detection method
5-LO	Arachidonic acid (25 µM)	Rhodamine 123	20 min / rt	Fluorimetry
12-LO	Arachidonic acid (4 µM)	Ferric oxidation of xylenol orange	5 min / rt	Photometry
15-LO-2	Arachidonic acid (10 µM)	15S-HpETE	90 min / 30 min	Fluorometry

Method B. For determination of 5-LO products in intact neutrophils, the cells (5×10^6) were resuspended in 1 mL PGC buffer, preincubated for 15 min at 37 °C with β-lapachone or vehicle (0.1% DMSO), and incubated for 10 min at 37 °C with 2.5 µM Ca^{2+}-ionophore A23187 plus 20 µM AA. The reaction was stopped on ice by addition of 1 mL of methanol, 30 µL 1 N HCL, 500 µL PBS, and 200 ng prostaglandin B_1 were added, and the samples were subjected to solid phase extraction on C18-columns (100 mg, UCT, Bristol, PA, USA). 5-LO products (LTB_4, tr-LTB_4 isomers, and 5-HETE), were analyzed by RP-HPLC and quantities calculated on the basis of the internal standard PGB_1. Cysteinyl-LTs C_4, D_4 and E_4 were not detected (amounts were below detection limit), and oxidation products of LTB_4 were not determined.

For analysis of 5-LO product formation in corresponding homogenates, neutrophils were resuspended in PBS containing 1 mM EDTA for 5 min at 4 °C and sonicated (4 × 10 s, 4 °C). Homogenates, corresponding to 5×10^6 cells/mL, were incubated with the test compounds or vehicle (0.1% DMSO) with or without 1 mM DTT for 15 min at 4 °C, pre-warmed for 30 s at 37 °C, and the reaction was started by the addition of 2 mM $CaCl_2$ plus the indicated concentrations of AA (routinely 20 µM). The reaction was stopped after 10 min and the samples were analyzed as described for intact cells above.

Data are expressed as mean ± S.E.M. IC_{50} values were calculated from averaged measurements at 6 different concentrations of the compound by nonlinear regression using GraphPad Prism software (San Diego, CA) one site binding competition. Statistical evaluation of the data was performed by one-way ANOVA followed by a Tukey-Kramer post-hoc test for multiple comparisons respectively. A p value < 0.05 (*) was considered significant.

1.4.5 Phosphodiesterase 5 assay

Phosphodiesterase 5 (PDE5) assay was performed at Cerep, SA (Celle l'Evescault, France) on a fee-for-service basis (Ref 0204[39]), as described in Table S5.

Table S5. Phosphodiesterase assay protocol.

Assay	Substrate	Measured component	Incubation	Detection method
Inhibition	$[^3H]$cGMP + cGMP (1 µM)	$[^3H]$5'GMP	60 min / rt	Scintillation counting

1.4.6 Minimum inhibitory concentration (MIC) assays

Escherichia coli K12 and *Staphylococcus aureus* ATCC 25923 were grown overnight at 37 °C and re-inoculated in 24-well plates containing 2.5 mL of Luria Bertani medium (LB) to give an optical density of ~0.01 at 600 nm. Stock solutions of β-lapachone, and lapachol were prepared in DMSO (1 % final concentration) and added to the cell suspensions to obtain final concentrations between 5-210 µM. Wells containing a growth control (cell suspensions with 1 % DMSO) and a sterile media control were also prepared. The plates were incubated for 18 h at 37 ºC and 90 r.p.m.. The concentration of compound in the first well in the series that presented no sign of visible growth was reported as the MIC. The OD600 of the cultures was also measured. All the MIC wells were serially diluted in phosphate buffer saline (PBS) and plated onto LB agar plates. Growth was evaluated after 24 h of incubation at 37 °C to access the minimum bactericidal concentration (MBC).

1.4.7 Cancer cell assays

HL-60 cells were routinely cultured in RPMI medium supplemented with 10% FBS, 1% Pen-strep and 1% HEPES at 5×10^5 cells/mL. 5-LO overexpression is stimulated by initially starving the cells in medium with 1% FBS for at-least 2-3 passages followed by growing the cells in 1.5% DMSO for another 3-4 days.[40]

For intracellular staining of 5-LO, 10^6 cells were collected and concentrated in 50 mL PBS. Cells were fixed with 100 mL BD fix buffer while vortexing. Cells were thoroughly washed with PBS and permeabilized using Perm Buffer followed by one hour incubation with primary anti 5-LO antibody (AB376, Merck Millipore). After the required incubation time, cells were washed and incubated for another hour with goat anti-rabbit Alexa fluor 488 antibody. Cells are then washed and resuspended in PBS and acquired by LSRFortessa™ flow cytometer (BD Biosciences, USA) with a 488 nm laser, a 505 nm long-pass filter and a 530/30 nm band-pass filter (for FITC detection). Data was analyzed using the FlowJo software.

HL-60 cells with and without DMSO stimulation were seeded at a concentration of 5×10^5 cells/mL in a 96 well plate format. Cells were treated with varying concentration of β-lapachone for a time period of 48 hours. Cell death was analyzed using standard Alamar Blue assay. Data is represented after being normalized to the vehicle control.

2. Supplementary data and discussion

2.1 Analyses of compound databases

Natural products are enriched in substructures often found in nuisance compounds, such as "frequent hitters"[41] and the so-called "pan assay interference compounds" (PAINS),[3, 42-44] which may afford intractable assay readouts and attrition. Despite the structural alerts, there is robust evidence that natural products provide less promiscuous target engagement profiles compared to structural alert-free synthetic small molecules.[45] Indeed, it has been recently shown by Bajorath and co-workers that PAINS may also bind specifically to drug targets.[46] For example, the troublesome quinone and catechol moieties are commonly featured by ligands in complex to proteins, as surveyed in the protein data bank.[46] Moreover, the original PAINS study reported that 86 out of 370 quinones presented a "clean" profile,[47] suggesting that such compounds may be used as prototypes for medicinal chemistry programs. β-Lapachone may thus afford opportunities for drug discovery.

Besides the therapeutic potential of β-lapachone, we drew inspiration from Clemons and co-workers[45] who have shown that natural products are generally better starting points for optimization than synthetic small molecules due to decreased promiscuity. Interestingly, our analysis of the Clemons *et al.*[45] dataset shows that only 30% of natural products pass the rapid elimination of swill (REOS)[48] and PAINS filters, whereas 53% of the more promiscuity-prone synthetic molecules are structurally "clean". From substructural and 2D pharmacophore vantage points,[8] we found overlapping chemical spaces between REOS/PAINS-complying and violating chemical matter (Figure S1a). Moreover, the drug-likeness of approved drugs, "clean" fragments in ZINC and REOS/PAINS-hitting fragment-like natural products, including **1**, is highly distributed. Our data thus advocates drug-likeness as a poor metric for prioritizing chemical matter (Figure S1b). Furthermore, it suggests that one cannot rationalize the elimination of structurally "ugly" compounds from screening libraries as a general approach using the abovementioned filtering rules. Of note, we computed that only *ca.* 50% of approved drugs are fully compliant with these filters, *i.e.* proper hit validation rather than *a priori* exclusion of chemical entities from screening assays may be more appropriate in select cases to avoid missed research opportunities.

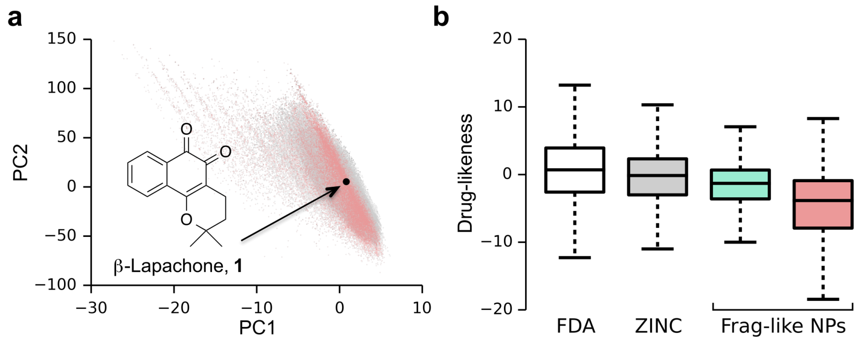

Figure S1. REOS/PAINS substructure-containing natural products (NPs) as potential leads for development. a) Principal component analysis for visualization of fragment-like REOS/PAINS-free chemical entities in ZINC15 (gray) and REOS/PAINS containing fragment–like NPs (red) using RDKit descriptors. b) Box plots of drug-likeness calculated with DataWarrior for FDA-approved drugs (white), REOS/PAINS-free fragment-like entities in ZINC15 (gray), REOS/PAINS-free fragment-like NPs (green) and REOS/PAINS-hitting

fragment-like NPs (red). Outliers were excluded. Drug-likeness is significantly different between approved drugs and fragment-like NP (two-sided Mann-Whitney test, $p < 0.0001$). Approved drugs: $n = 1506$; REOS/PAINS-free fragment-like NPs: $n = 35376$; REOS/PAINS-hitting fragment-like NPs: $n = 35544$.

2.2 Target prediction

We carried out drug target predictions with SPiDER. Importantly, known targets (*e.g.* DNA topoisomerase,[49] cyclooxygenase[50]) were predicted retrospectively, advocating for the appropriateness of our approach, while other methods underperformed in this particular case (Table S6-11).

Table S6. Target predictions for β-lapachone with SPiDER (p values < 5%).

**Target family**	***p* value %**
DNA topoisomerase	0.0
Nicotinic acetylcholine receptor	0.1
11β-Hydroxysteroid dehydrogenase	0.2
Interleukin receptor	0.2
TNFα	0.3
Androgen receptor	0.4
Cyclooxygenase	0.7
Ionotropic glutamate receptor	0.8
5-Lipoxygenase	1.0
Phosphodiesterase	1.1
Potassium channel	1.2
Metabotropic glutamate receptor	1.2
Glucagon-like peptide receptor	1.2
Monoamine oxidase	1.6
Serotonin receptor	1.6
Prostanoid receptor	1.7
Acetylcholinesterase	1.8
Peroxisome proliferator-activated rceptor	1.8
Sodium neurotransmitter symporter	1.9
Nicotinicoid GABA receptor	2.2
Adenosine receptor	2.4
Integrins	2.5
Transient receptor potential channel	2.6
Serine threonine kinase	3.1
Tyrosine kinase	3.6
Cannabinoid receptor	3.8

Table S7. Target predictions for lapachol with SPiDER (p values $< 5\%$).

**Target family**	***p* value %**
5-Lipoxygenase	1.5
Histone deacetylase	2.4
Prostanoid receptor	2.9
Endopeptidase	3.0
Aldose reductase	3.1
Peroxisome proliferator-activated receptor	3.2
Metabolotropic glutamate receptor	3.4
Serine threonine kinase	3.6
Sodium channel	3.7
Aromatase	4.1
Phosphodiesterase	4.2
Aggregation inhibitor	4.5
Phosphoinositide kinase	4.9

Table S8. Confident target predictions for β-lapachone with SEA.

**Target family**	**E-value**
Indoleamine 2,3-dioxygenase	5.55e-12

Table S9. Confident target predictions for lapachol with SEA.

**Target family**	**E-value**
DNA lyase	2.15e-53
Leucocyte common antigen	2.38e-46
Glutathione reductase	2.73e-41
Protein-tyrosine phosphatase 1E	2.78e-28

Table S10. Target predictions for β-lapachone with SuperPred.

**Target family**	**E-value**
Indoleamine 2,3-dioxygenase	2.25e0

Table S11. Confident target predictions for lapachol with SuperPred.

**Target family**	**E-value**
G-protein coupled receptor 35	4.38e-1
Sentrin-specific protease 6	2.92e0

2.3 Kinase, ion channel and enzyme screening

With drug target predictions in hand, we then screened β-lapachone and its isomer lapachol at 150 µM, *i.e.* where 50% effect equates to a ligand efficiency of 0.30 (Tables S12-16). Given the potent effects observed against 5-LO, we confirmed inhibition in concentration response curves using cell-free assays with two different detection methods: i) direct measurement of 5-LO reaction products and ii) indirect fluorescence detection method (Cerep, France). In the latter case, β-lapachone potently inhibited 5-LO (IC_{50} = 2.1 µM ± 0.23 log units, $n = 2$; $LE = 0.44$; Figure S2). No interference from auto-fluorescence of β-lapachone was detected in the 5-LO assay, corroborating the data obtained for the first method.

Table S12. Kinase panel screening for β-lapachone at 150 µM ($n = 2$).

Kinase	% Inhibition of control values			Flag
	1st measurement	2nd measurement	Mean	
EGFR	68.5	65.7	**67.1**	
AurA	-16.5	0.2	-8.1	Interference
IKKα	59.5	59.4	**59.5**	Interference
NEK2	19.0	18.4	18.7	Interference
PLK1	61.1	58.9	**60.0**	
c-Met	45.3	50.6	47.9	Interference
EphA2	63.7	63.5	**63.6**	Interference
EphA3	66.8	63.3	**65.1**	
EphB4	54.6	55.1	**54.8**	Interference
FGFR1	51.5	60.5	**56.0**	Interference
FGFR3	76.5	85.1	**80.8**	Interference
FGFR2	44.8	55.2	**50.0**	Interference
IRK	50.9	50.6	**50.8**	
VEGFR2	82.7	82.0	**82.3**	
TRKA	38.1	45.7	41.9	Interference
JAK3	83.6	54.5	**69.0**	
Lck	-32.6	-37.7	-35.2	Interference
Src	36.1	39.5	37.8	
CDK1	66.0	68.0	**67.0**	Interference
CDK2	79.4	77.1	**78.2**	
ERK2	44.8	46.9	45.9	
GSK3β	95.0	93.4	**94.2**	
JNK1	59.8	55.6	**57.7**	Interference
p38α	27.1	23.1	25.1	
CaMK2α	61.5	48.0	**54.8**	
CHK1	9.4	19.7	14.5	Interference
CHK2	46.6	59.5	**53.1**	Interference
MAPKAPK2	-14.5	-2.7	-8.6	Interference
Abl	42.9	41.8	42.4	
MARK1	-70.1	-44.7	-57.4	
MNK2	78.1	82.1	**80.1**	
Pim2	79.6	82.6	32.5	Interference

SIK	41.8	40.3	41.0	Interference
Akt1/PKBα	65.8	55.0	**60.4**	Interference
PKA	-10.5	-5.4	-8.0	Interference
PDK1	8.9	-6.1	1.4	
PKCβ2	36.5	31.6	34.0	
ROCK1	-12.5	-3.2	-7.9	Interference
SGK1	54.4	54.5	**54.5**	
MAP4K4	47.7	49.5	48.6	
PAK2	-34.0	1.5	-16.2	Interference
PAK4	-3.7	0.0	-1.9	
TAOK2	32.9	33.1	33.0	Interference
IRAK4	-1.2	5.6	2.2	
RAF-1	55.4	59.3	**57.3**	

Controls – Abl: Staurosporine $IC_{50} = 2.0\times10^{-7}$M (*nHill* = 1.3); EGFR: PD153035 $IC_{50} = 3.8\times10^{-10}$M (*nHill* = 0.9); AurA: Staurosporine $IC_{50} = 2.3\times10^{-8}$M (*nHill* = 2.5); IKKα: Staurosporine $IC_{50} = 7.9\times10^{-8}$M (*nHill* = 1.7); NEK2: Staurosporine $IC_{50} = 1.0\times10^{-6}$M (*nHill* = 0.9); PLK1: Staurosporine $IC_{50} = 1.1\times10^{-6}$M (*nHill* = 1.1); c-Met: Staurosporine $IC_{50} = 1.4\times10^{-7}$M (*nHill* = 1.8); EphA2: Staurosporine $IC_{50} = 4.0\times10^{-7}$M (*nHill* = 1.6); EphA3: Staurosporine $IC_{50} = 8.1\times10^{-8}$M (*nHill* = 0.9); EphB4: Staurosporine $IC_{50} = 7.8\times10^{-7}$M (*nHill* = 1.1); FGFR1: Staurosporine $IC_{50} = 2.5\times10^{-8}$M (*nHill* = 1.2); FGFR3: Staurosporine $IC_{50} = 9.6\times10^{-9}$M (*nHill* = 1.0); FGFR2: Staurosporine $IC_{50} = 9.0\times10^{-9}$M (*nHill* >3); IRK: Staurosporine $IC_{50} = 4.0\times10^{-8}$M (*nHill* = 1.0); VEGFR2: Staurosporine $IC_{50} = 4.2\times10^{-9}$M (*nHill* = 1.2); TRKA: Staurosporine $IC_{50} = 9.5\times10^{-9}$M (*nHill* >3); JAK3: Staurosporine $IC_{50} = 2.2\times10^{-9}$M (*nHill* = 2.2); Lck: Staurosporine $IC_{50} = 5.1\times10^{-8}$M (*nHill* = 1.3); Src: Staurosporine $IC_{50} = 5.5\times10^{-9}$M (*nHill* = 1.0); CDK1: Staurosporine $IC_{50} = 2.9\times10^{-8}$M (*nHill* = 1.1); CDK2: Staurosporine $IC_{50} = 6.5\times10^{-9}$M (*nHill* = 1.4); ERK2: Staurosporine $IC_{50} = 7.2\times10^{-7}$M (*nHill* = 1.1); GSK3β: Staurosporine $IC_{50} = 6.0\times10^{-8}$M (*nHill* = 1.6); JNK1: Staurosporine $IC_{50} = 1.1\times10^{-6}$M (*nHill* = 1.8); p38α: SB202190 $IC_{50} = 5.7\times10^{-8}$M (*nHill* = 1.3); CaMK2α: AIP $IC_{50} = 1.7\times10^{-7}$M (*nHill* = 0.8); CHK1: Staurosporine $IC_{50} = 6.7\times10^{-9}$M (*nHill* = 1.5); CHK2: Staurosporine $IC_{50} = 2.6\times10^{-8}$M (*nHill* = 1.9); MAPKAPK2: Staurosporine $IC_{50} = 4.6\times10^{-7}$M (*nHill* = 1.6); MARK1: Staurosporine $IC_{50} = 7.2\times10^{-9}$M (*nHill* = 2.5); MNK2: Staurosporine $IC_{50} = 3.1\times10^{-8}$M (*nHill* >3); Pim2: Staurosporine $IC_{50} = 3.8\times10^{-8}$M (*nHill* = 1.5); SIK: Ro-318220 $IC_{50} = 1.8\times10^{-8}$M (*nHill* = 0.7); Akt/PKBα: Staurosporine $IC_{50} = 2.9\times10^{-8}$M (*nHill* = 0.8); PKA: Staurosporine $IC_{50} = 1.0\times10^{-8}$M (*nHill* >3); PDK1: Staurosporine $IC_{50} = 5.0\times10^{-8}$M (*nHill* >3); PKCβ2: Staurosporine $IC_{50} = 4.9\times10^{-9}$M (*nHill* = 1.1); ROCK1: Staurosporine $IC_{50} = 2.6\times10^{-8}$M (*nHill* = 2.7); SGK1: Staurosporine $IC_{50} = 3.4\times10^{-8}$M (*nHill* >3); MAP4K4: Staurosporine $IC_{50} = 3.7\times10^{-8}$M (*nHill* = 2.7); PAK2: Staurosporine $IC_{50} = 3.2\times10^{-8}$M (*nHill* >3); PAK4: Staurosporine $IC_{50} = 6.6\times10^{-8}$M (*nHill* = 3.0); TAOK2: Staurosporine $IC_{50} = 5.4\times10^{-8}$M (*nHill* = 1.6); IRAK4: Staurosporine $IC_{50} = 3.7\times10^{-8}$M (*nHill* = 0.7); RAF1: Staurosporine $IC_{50} = 2.6\times10^{-7}$M (*nHill* = 2.4).

Table S13. EP1-4 screening for β-lapachone and lapachol at 150 µM ($n = 2$).

GPCR (effect)	Compound	% of control values			Flag
		1st measurement	2nd measurement	Mean	
EP1 (agonist)	β-Lapachone	-11.9	-11.9	-11.9	
	Lapachol	-11.3	-11.3	-11.3	
EP1 (antagonist)	β-Lapachone	28.6	28.5	28.6	
	Lapachol	22.5	23.7	23.1	
EP2 (agonist)	β-Lapachone	12.3	14.8	13.5	
	Lapachol	-1.2	-2.4	-1.8	
EP2 (antagonist)	β-Lapachone	59.7	60.7	**60.2**	
	Lapachol	34.7	3.3	19.0	
EP3 (agonist)	β-Lapachone	18.6	15.5	17.0	
	Lapachol	22.7	21.7	22.2	
EP3 (antagonist)	β-Lapachone	96.1	93.6	**94.9**	
	Lapachol	47.7	51.4	49.5	
EP4 (agonist)	β-Lapachone	54.6	51.7	**53.1**	
	Lapachol	8.0	5.8	6.9	
EP4 (antagonist)	β-Lapachone	-57.2	-68.4	-62.8	
	Lapachol	63.0	48.8	**55.9**	

Controls – EP_1 functional agonist: PGE_2 EC_{50} = 6.9×10^{-10}M; EP_1 functional antagonist: SC51322 IC_{50} = 1.2×10^{-8}M; EP_2 functional agonist: PGE_2 EC_{50} = 1.6×10^{-8}M; EP_2 functional antagonist: AH 6809 IC_{50} = 2.8×10^{-6}M; EP_3 functional agonist: Sulprostone EC_{50} = 2.3×10^{-11}M; EP_3 functional antagonist: L798106 IC_{50} = 3.7×10^{-7}M; EP_4 functional agonist: PGE_2 EC_{50} = 1.2×10^{-9}M; EP_4 functional antagonist: GW627368X IC_{50} = 2.0×10^{-8}M.

Table S14. TRP channel screening for β-lapachone and lapachol at 150 µM ($n = 2$).

TRP (effect)	Compound	% of control values			Flag
		1st measurement	2nd measurement	Mean	
TRPV1 (agonist)	β-Lapachone	-7.3	-1.9	-4.6	
	Lapachol	-13.1	-13.1	-13.1	
TRPV1 (antagonist)	β-Lapachone	70.0	56.5	**63.2**	
	Lapachol	67.0	63.6	**65.3**	
TRPM8 (agonist)	β-Lapachone	-20.4	-20.4	-20.4	
	Lapachol	-20.4	-20.4	-20.4	
TRPM8 (antagonist)	β-Lapachone	41.5	36.0	38.8	
	Lapachol	89.7	92.0	**90.8**	

Controls – TRPV1 functional agonist: Capsaicin EC_{50} = 3.4×10^{-9}M; TRPV1 functional antagonist: Capsazepin IC_{50} = 9.3×10^{-8}M; TRPM8 functional agonist: Icilin EC_{50} = 1.7×10^{-8}M; TRPM8 functional antagonist: BCTC IC_{50} = 2.6×10^{-7}M.

Table S15. PDE5 screening for β-lapachone at 2 µM ($n = 2$).

% of control values			Flag
1st measurement	2nd measurement	Mean	
-6.9	-9.7	**-8.3**	

Control – Dipyridamole IC_{50} = 1.6×10^{-6}M (*nHill* = 1.3).

Table S16. 5-LO, 12-LO and 15-LO-2 screening for β-lapachone, lapachol and analogues (n = 2).

Cpd	Enzyme	Conc. / µM	% inhibition of control values			Flag
			1st measure	2nd measure	Mean	
β-Lapachone	5-LO	150	98.4	92.9	**95.7**	
Lapachol	5-LO	150	84.9	89.3	**87.1**	
β-Lapachone	5-LO	5	103.2	99.8	**101.5**	
2	5-LO	5	42.2	22.2	32.2	
3	5-LO	5	33.1	29.0	31.1	
4	5-LO	5	77.4	68.2	**72.8**	
5	5-LO	5	47.2	35.5	41.1	
6	5-LO	5	51.5	58.1	**54.8**	
7	5-LO	5	73.8	76.9	**75.4**	
8	5-LO	5	68.4	64.8	**66.6**	
β-Lapachone	12-LO	5	40.7	35.3	38.0	
β-Lapachone	15-LO-2	5	-12.0	-13.8	-12.9	

Controls – 5-LO: NDGA IC_{50} = 1.2×10^{-7}M (*nHill* = 1.5); 12-LO: NDGA IC_{50} = 1.3×10^{-6}M (*nHill* = 1.8); 15-LO-2: 2-TEDC IC_{50} = 3.3×10^{-6}M (*nHill* = 0.7).

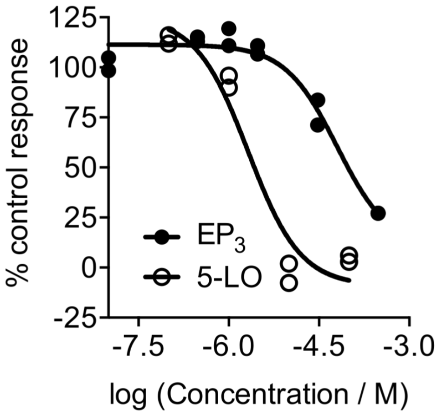

Figure S2. Concentration–inhibition curves of 5-LO and EP3 by β-lapachone. EP_3: IC_{50} = 63 µM ± 0.15 log units; K_B = 22 µM (*LE* = 0.36); n = 2 (control: L798106; IC_{50} = 0.37 µM). 5-LO: IC_{50} = 2.1 µM ± 0.23 log units (*LE* = 0.44); n = 2 (control: NDGA; IC_{50} = 0.12 µM, nHill = 1.5).

From a cheminformatics vantage point, the result is also important as β-lapachone is only scarcely related on a substructural level to known 5-LO inhibitors on average. The most related 5-LO inhibitor presents a Tanimoto index < 0.30, which supports structural dissimilarity to β-lapachone (Figure S3a) and that the ortho-quinone scaffold is not exploited as motif in 5-LO inhibitors. Conversely, analyzing topological pharmacophores, the conclusion is opposed, as β-lapachone lies in a region populated by other 5-LO inhibitors (Figure S3b) and providing a rationale for testing of β-lapachone against 5-LO.

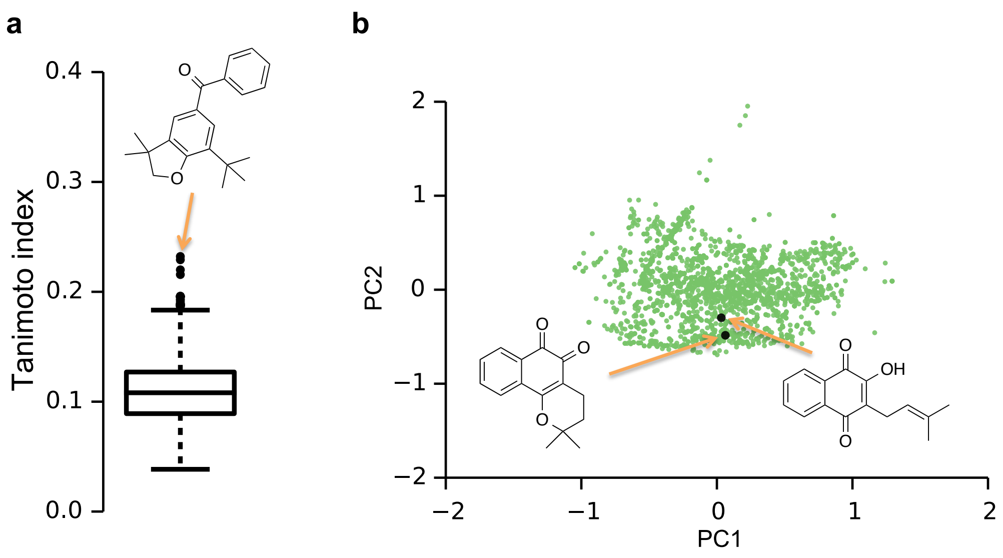

Figure S3. ChEMBL22 5-lipoxygenase inhibitor space. a) Nearest neighbor to β-lapachone as computed with ECFP4-like Morgan fingerprints, radius 2, 2048 bits. Nearest neighbor (CHEMBL275120, Tanimoto index = 0.23) is shown. b) Principal component analysis of 5-lipoxygenase inhibitor space together with β-lapachone and lapachol, using the pharmacophore autocorrelation descriptor CATS as implemented in MOE 2015.10.

Testing of β-lapachone against human neutrophils showed selectivity for 5-LO, over 12- and 15-LO (Figure S4a), albeit with lower potency compared to the cell-free 5-LO inhibition assays. Our data show that β-lapachone must be converted to the hydroquinone form for potent 5-LO modulation. A possible explanation for the obtained lower potency might be the insufficient conversion in the native neutrophil environment. However, supplementing neutrophils with dithiothreitol reinstates potency similar to that obtained in cell free assays, which corroborates our hypothesis (Figure S4b).

Overall, the obtained cell-free and whole cell data was reproducible with three different sources of β-lapachone – two different synthetic routes devised by us, and one commercial (Bide Pharmatech Ltd).

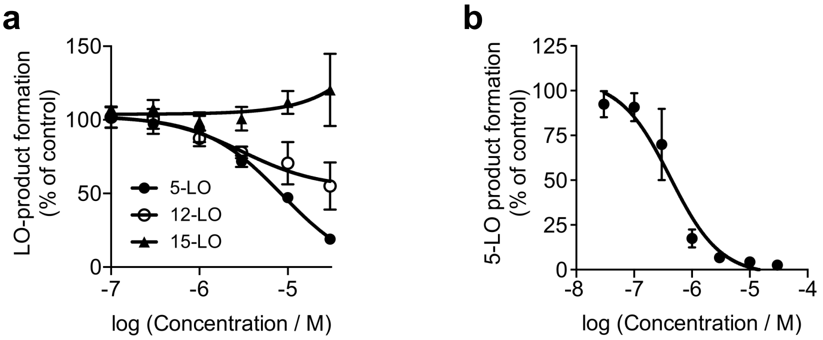

Figure S4. Biochemical profile of β-lapachone. a) Inhibition of 5-, 12- and 15-LO in intact human neutrophils (without DTT). IC_{50} (5-LO) = 8.6 µM ± 0.10 log units, n = 3; IC_{50} (12-LO) > 30 µM; IC_{50} (15-LO) > 30 µM. b) Inhibition of 5-LO activity in intact human neutrophils supplemented with 1 mM DTT. IC_{50} = 0.42 µM ± 0.11 log units, n = 4.

2.4 β-Lapachone-inspired chemical library

To further validate binding and engagement of 5-LO by β-lapachone we built a focused library to probe not only structure–activity relationships but also ascertain a non-flat bioactivity landscape which could be considered a flag for unspecific binding (Figure 3a). Generally, the *in situ* bromination of an appropriate starting material afforded the respective key intermediates, which were subsequently functionalized with the required nucleophilic species.[20, 23, 26] A range of inhibition potencies were obtained in cell-free 5-LO assays for compounds **2–8** (Figure 3a), supporting the importance of the substitution pattern for bioactivity and the specific, directed interactions of **1** with 5-LO. For example, the activity of the β-lapachone-inspired entities against 5-LO appears to be sensitive to ring contraction (*e.g.* β-lapachone *vs.* **4**), stereogenic centre configuration (β-lapachone *vs.* **2–8**) and potentially to desolvation/thermodynamics penalties (β-lapachone *vs.* **5**). Although not probed in this study it is conceivable that (*R*)- and (*S*)-configured molecules present different binding affinities to 5-LO. Molecular docking of (*R*)-**4** and (*S*)-**4**, indeed suggests that diverging configurations can impact molecular recognition and bioactivity against 5-LO (Figure S5).

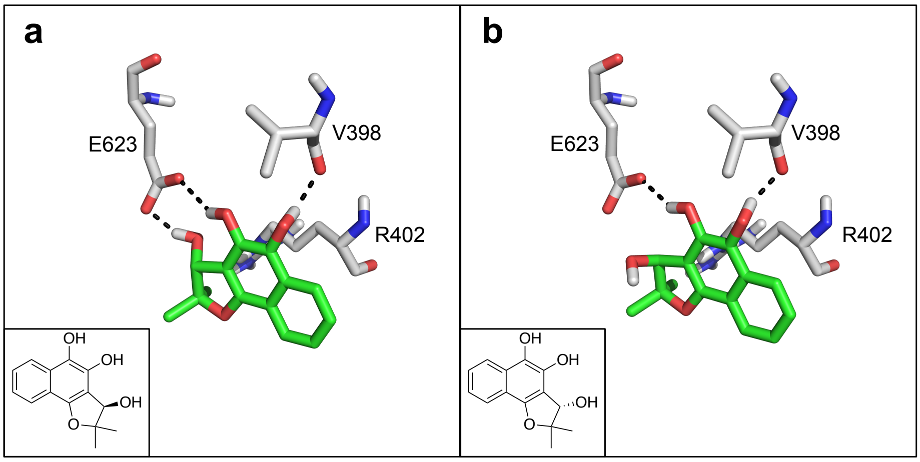

Figure S5. Molecular docking poses of (*R*)-**4** (panel a) and (*S*)-**4** (panel b) at the predicted 5-LO binding pocket.

2.5 Anti-microbial screening

As counter screen, we next tested β-lapachone against *Escherichia coli* and *Staphylococcus aureus*, given their sensitivity to oxidative stress induced by reactive oxygen species-producing chemicals.[51-54] No phenotypic effects were observable at concentrations up to 50 µM of β-lapachone (*cf.* Figure S6). Together, our data supports the absence of general target modulation by β-lapachone.

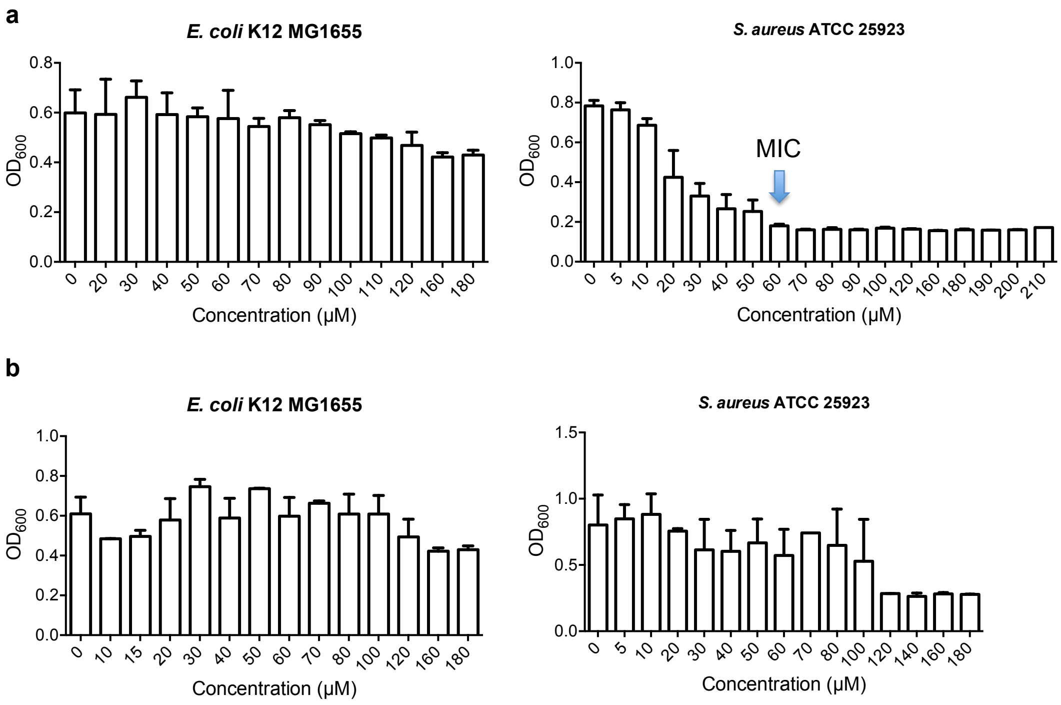

Figure S6. Screening of β-lapachone (a) and lapachol (b) against gram positive (*Staphylococcus aureus*) and gram negative (*Escherichia coli*) bacteria.

2.6 Mechanism of 5-LO inhibition by β-lapachone

To understand the molecular mechanism of 5-LO inhibition by β-lapachone we carried out wash-out experiments (Figure S7a) to probe the reversibility of the binding interaction. In addition, because β-lapachone presents the structural requirements to chelate active site iron, we performed competition assays with the natural substrate arachidonic acid. Our results show that binding of β-lapachone is non-competitive, suggesting an allosteric modulation mechanism (Figure S7b). Altogether we present evidence that β-lapachone requires to be reduced to its hydroquinone form (*e.g.* through NQO1 in cancer cells) in order to modulate 5-LO (Figure S7c). Naturally, several mechanisms of anticancer activity may come into play. β-Lapachone may itself modulate hitherto unknown drug targets while the generated reactive oxygen species in the redox cycle are also accountable for the phenotypic effects.

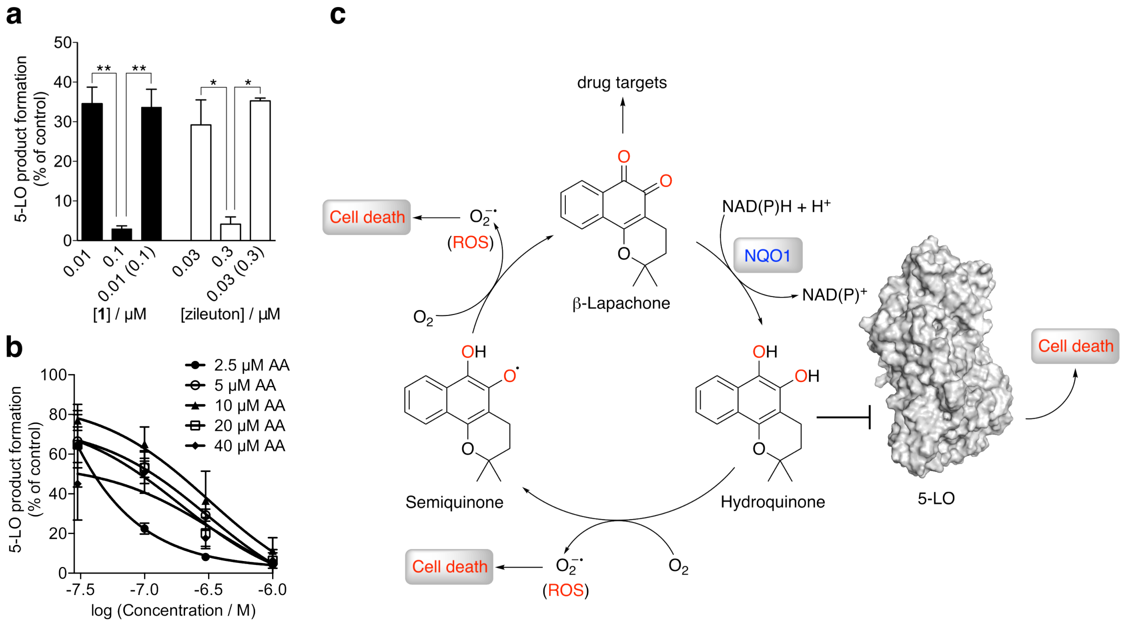

Figure S7. Proposed mechanism of action of β-lapachone, **1**. a) Wash-out experiments with purified 5-lipoxygenase (5-LO) pre-incubated with 0.01 or 0.1 µM of **1**, 0.03 or 0.3 µM zileuton, or vehicle (0.1% DMSO) plus 1 mM DTT, each. Samples containing either 0.1 µM of **1** or 0.3 µM zileuton were then diluted 10-fold. After addition of 2 mM $CaCl_2$, 20 µM of arachidonic acid (AA) and 10 min incubation, 5-LO product formation was determined, n = 3. Statistics: one-way ANOVA followed by a Tukey-Kramer post-hoc test for multiple comparisons; $*p < 0.05$; $**p < 0.01$. b) Inhibition of 5-LO by **1** at varying AA concentrations. Data show non-competitive inhibition, n = 3. c) Activation of the quinone results in a hydroquinone species responsible for generation of reactive oxygen species (ROS) and for inhibition of 5-LO.

Indeed, β-lapachone binds to 5-LO competitively to phosphatidylcholine, which is known to increase the catalytic activity of 5-LO (Figure S8). The predicted binding pose suggests no interaction with tryptophan residues. Because tryptophan residues display fluorescence under certain experimental conditions, we challenged our binding model by monitoring tryptophan fluorescence; wherein a blue shift indicates a binding interaction. Using purified human 5-LO and supplementing it with 1 mM dithiothreitol to ensure reduction of β-lapachone to the corresponding hydroquinone we observed no shift in fluorescence at relevant binding concentrations (Figure S9). Hence, one may conclude that tryptophan residues do not intervene in the binding event of β-lapachone to 5-LO and that at concentrations as high as 10 µM of β-lapachone no denaturation of 5-LO occurs.

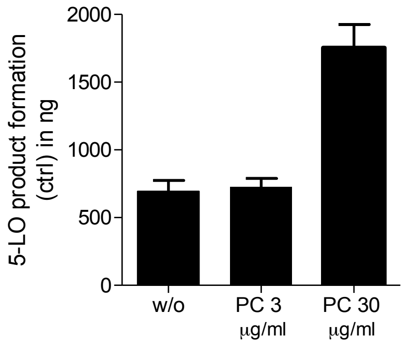

Figure S8. 5-Lipoxygenase product formation with increasing amounts of phosphatidylcholine (PC).

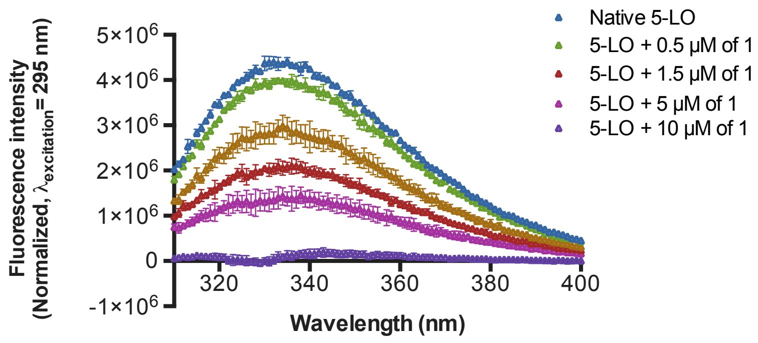

Figure S9. Tryptophan fluorescence as a function of β-lapachone, **1**, concentration.

2.7 Anticancer assays

To assess the importance of modulation of a given target in cancer, the model system most commonly employed is the use of siRNA to suppress expression of the gene of interest and then assess cell survival in the silenced population. A statistically significant difference in cell viability between the gene-silenced cells and the wild type cells usually provides initial proof-of-concept. The method however suffers from caveats: i) silencing is often not very efficient, being common to find only 60-70% of cells not expressing the protein of interest or with reduced expression; ii) cell viability may be deeply affected by silencing genes. Herein we used the approach of overexpressing 5-LO. While transfection still suffers from identical success rates, through this approach one does not shut down potentially critical pathways but instead exacerbates them. Thus, cells with exacerbated activity for a given protein will be more sensitive to modulators if: i) the target is important for cancer cell survival and ii) if inhibition of the protein of interest is relevant for the anticancer activity of the studied molecule. A similar approach has already been successfully followed to study DYRK3 biology.[55]

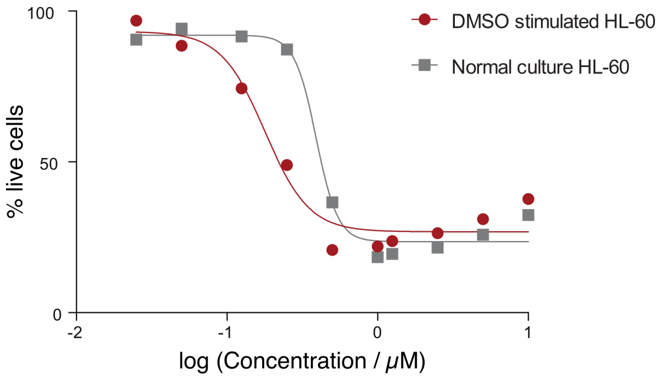

Figure S10. IC_{50} curves for β-lapachone against viability of the leukemia HL-60 cell line.

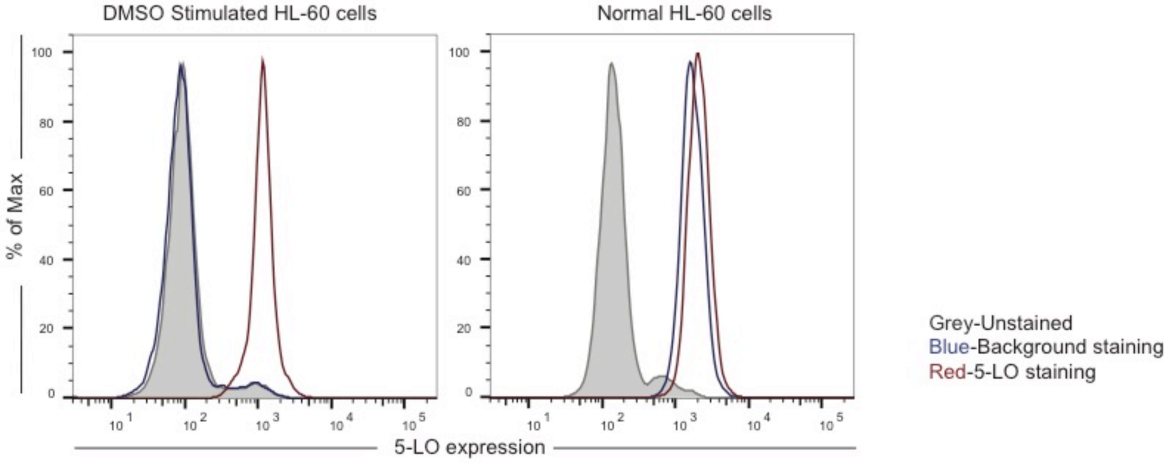

Figure S11. Fluorescence-assisted cell sorting (FACS) data. Intracellular 5-LO was detected in both cell lines. In normal HL-60 cells, the background staining is relatively high and the shift upon 5-LO staining overlaps with the background making it a non-specific staining.

2.8 Spectral data

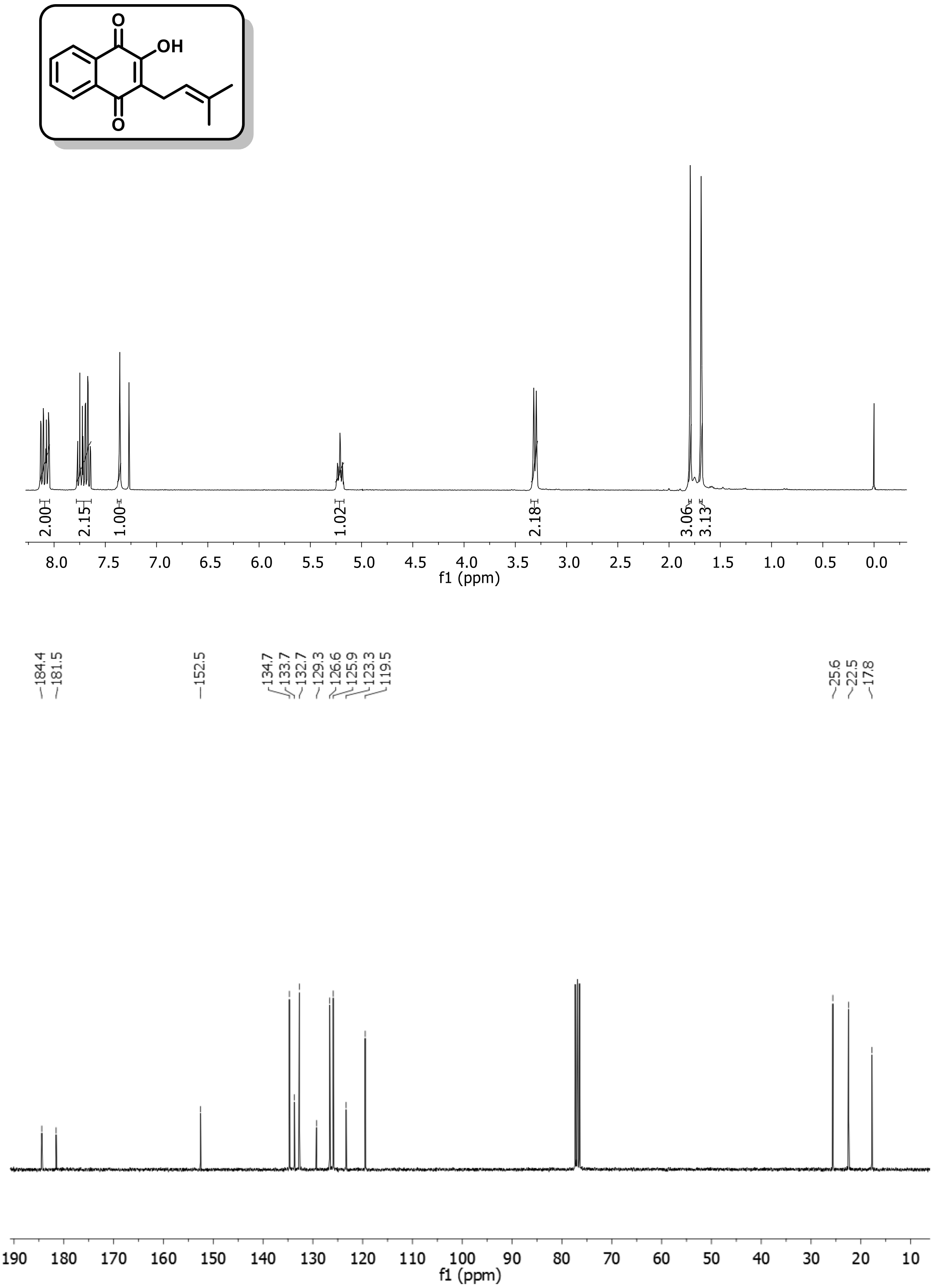

Figure S12. ^{1}H and ^{13}C NMR spectra of lapachol recorded in $CDCl_3$ (300 K).

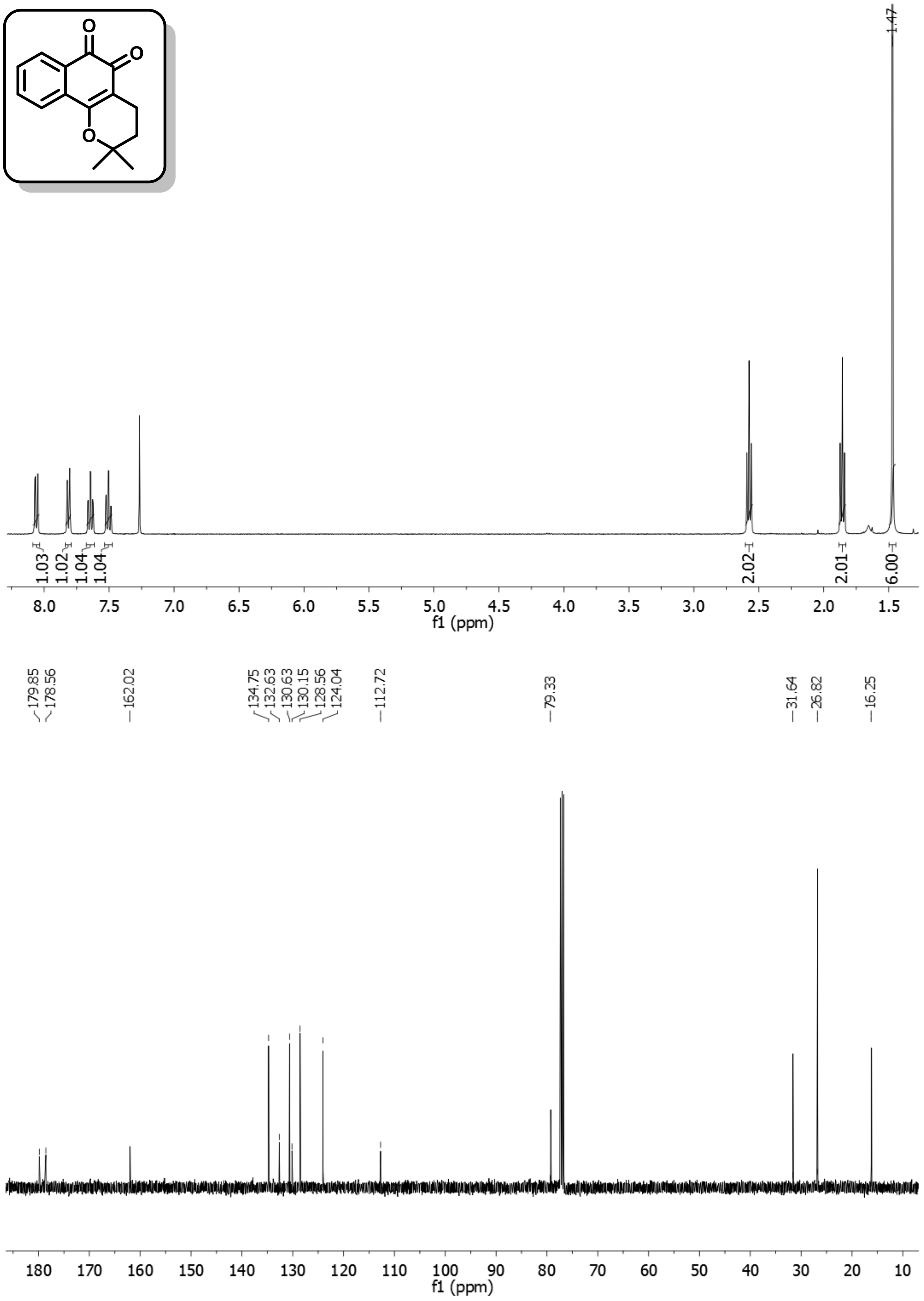

Figure S13. ^{1}H and ^{13}C NMR spectra of β-lapachone recorded in $CDCl_3$ (300 K).

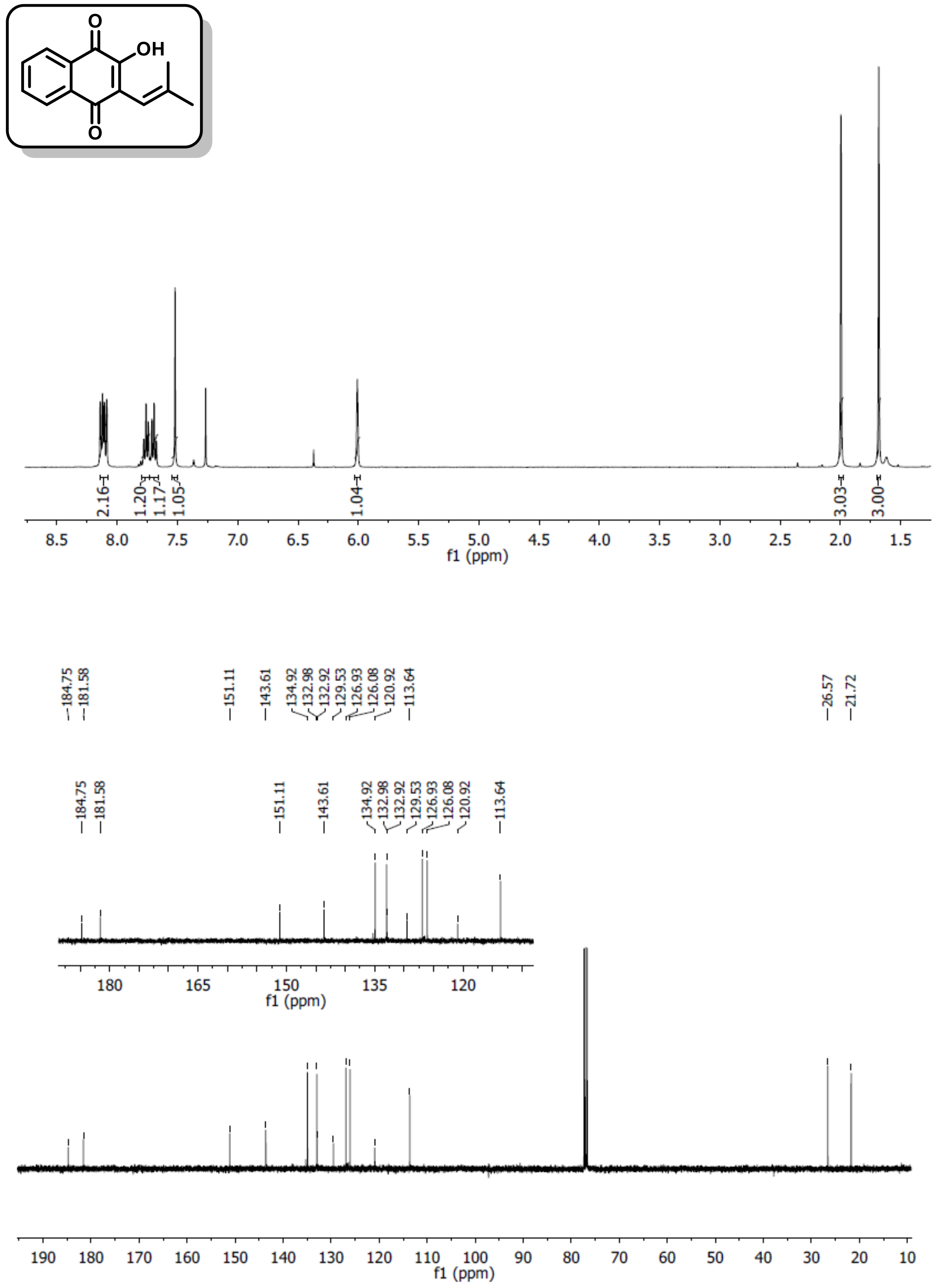

Figure S14. ^{1}H and ^{13}C NMR spectra of nor-lapachol recorded in $CDCl_3$ (300 K).

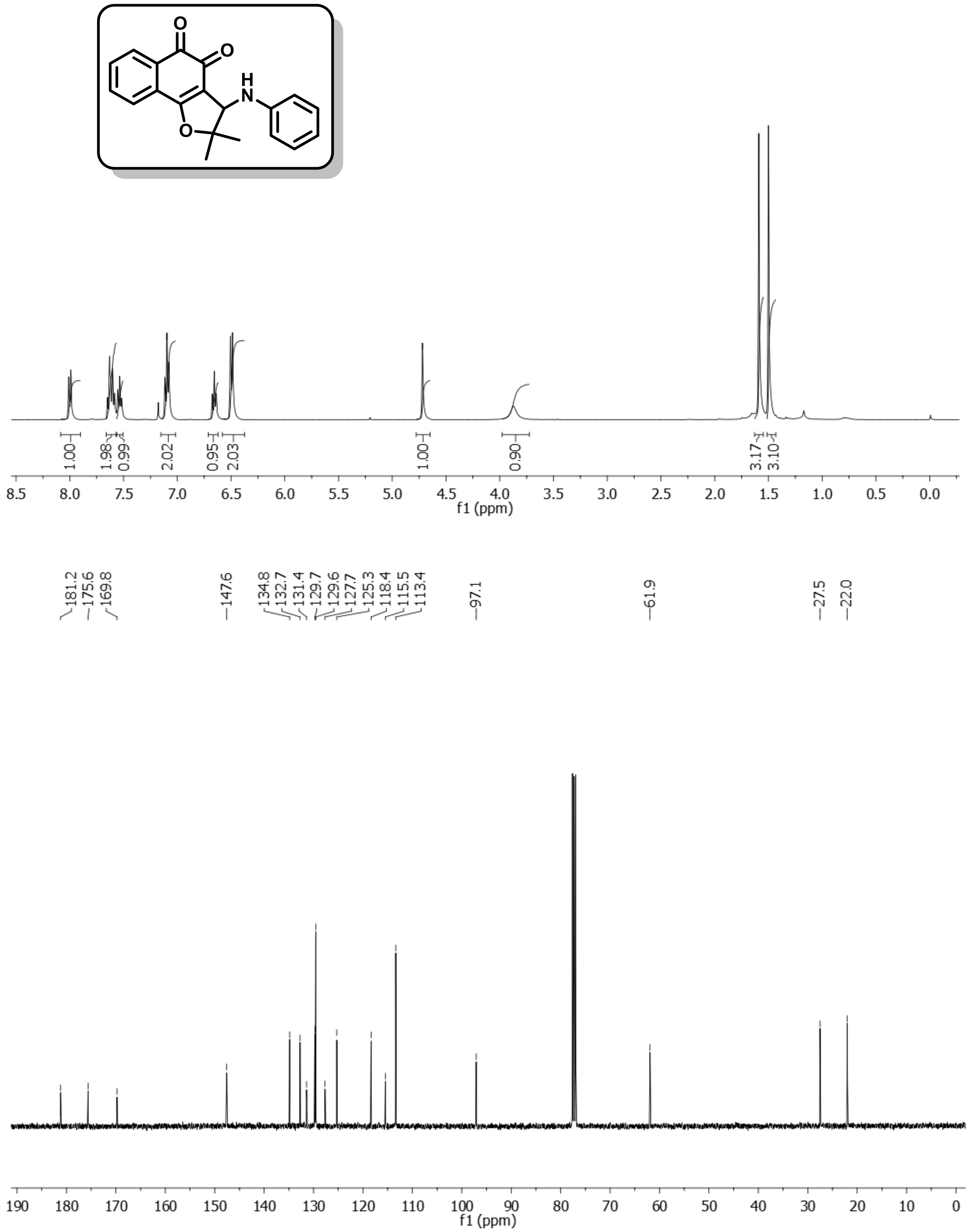

Figure S15. ^{1}H and 13C NMR spectra of 3-arylamino-nor-β-lapachone at 400 MHz in $CDCl_3$ (300 K).

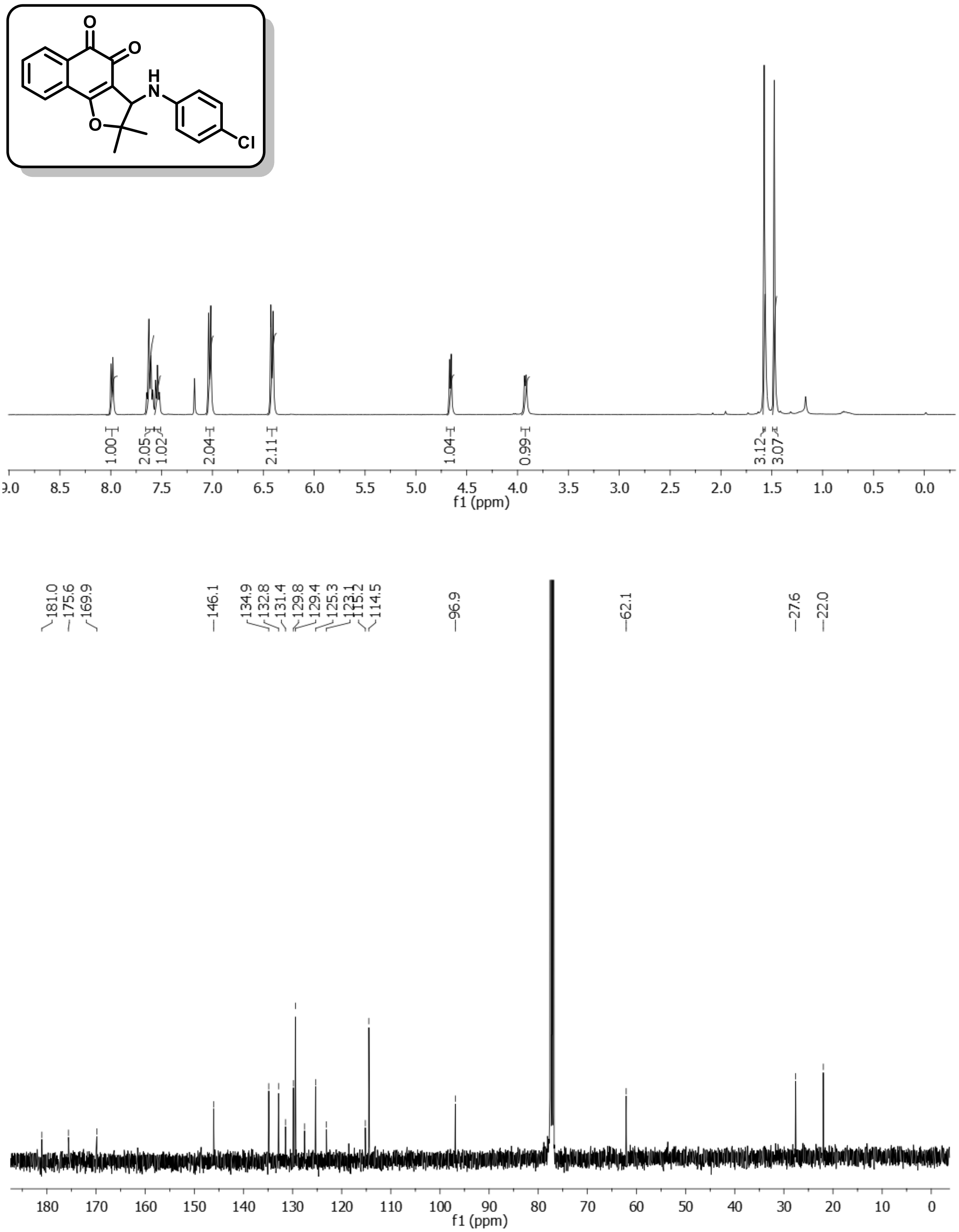

Figure S16. ^{1}H and 13C NMR spectra of *p*-chloro-3-arylamino-nor-β-lapachone at 400 MHz in $CDCl_3$ (300 K).

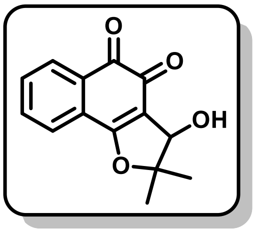

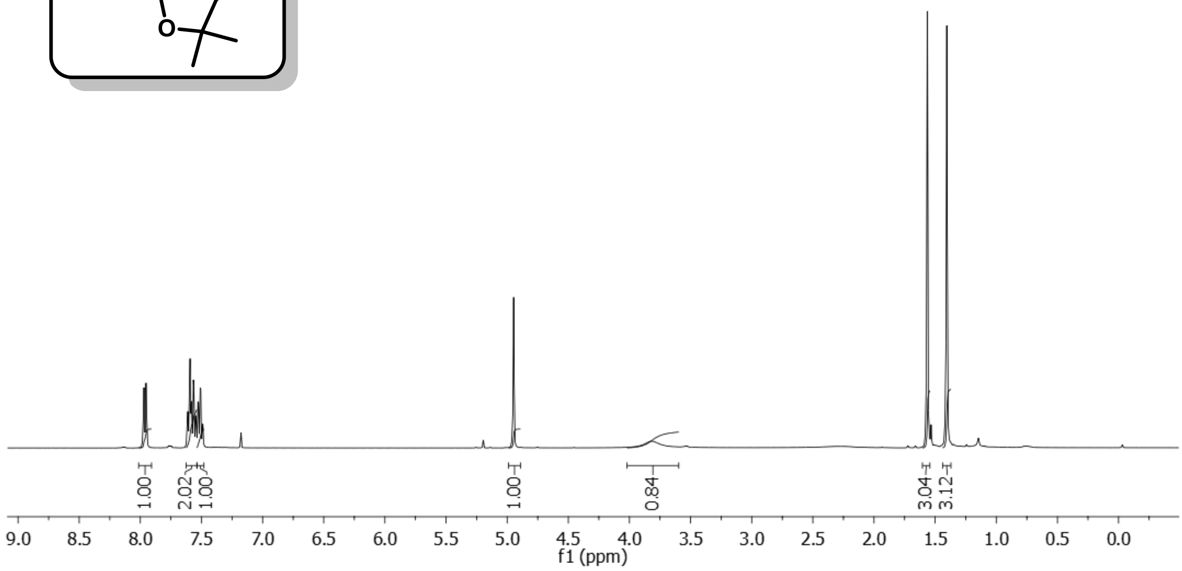

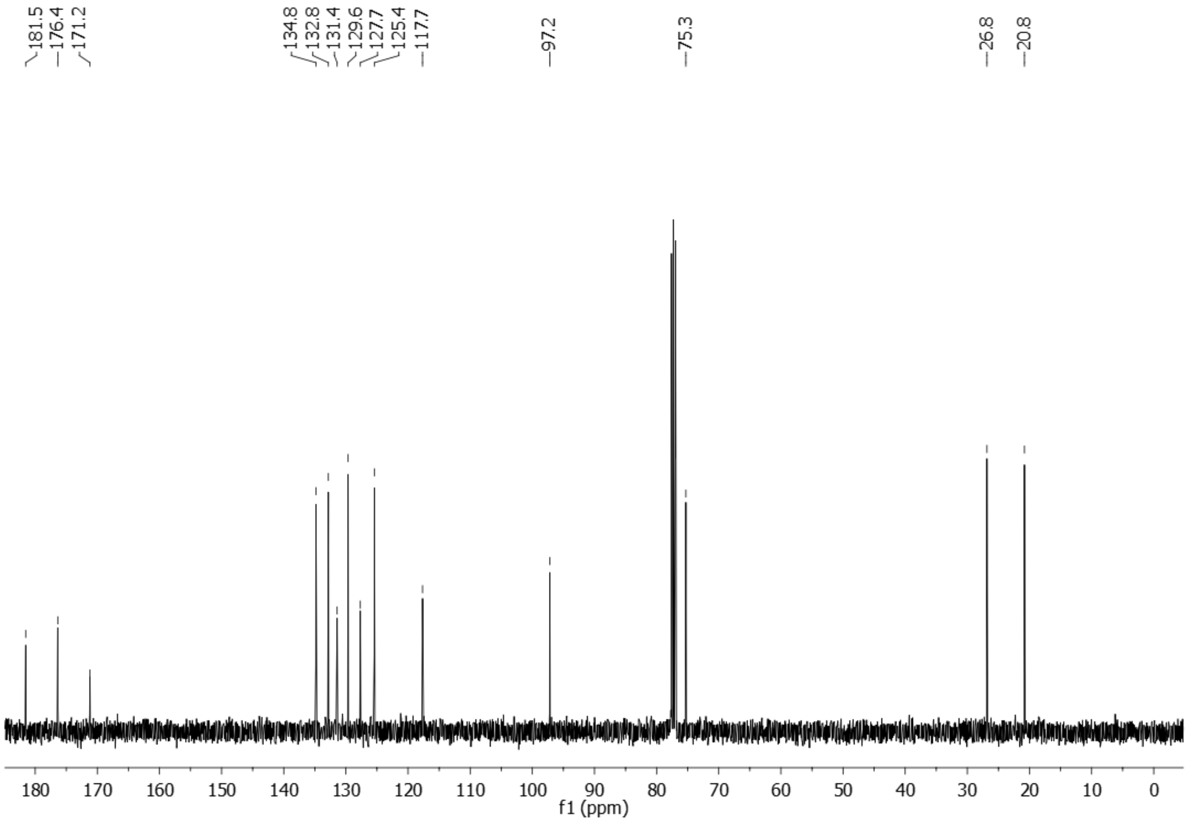

Figure S17. ^{1}H and ^{13}C NMR spectrum of 3-hydroxy-nor-β-lapachone at 400 MHz in $CDCl_3$ (300 K).

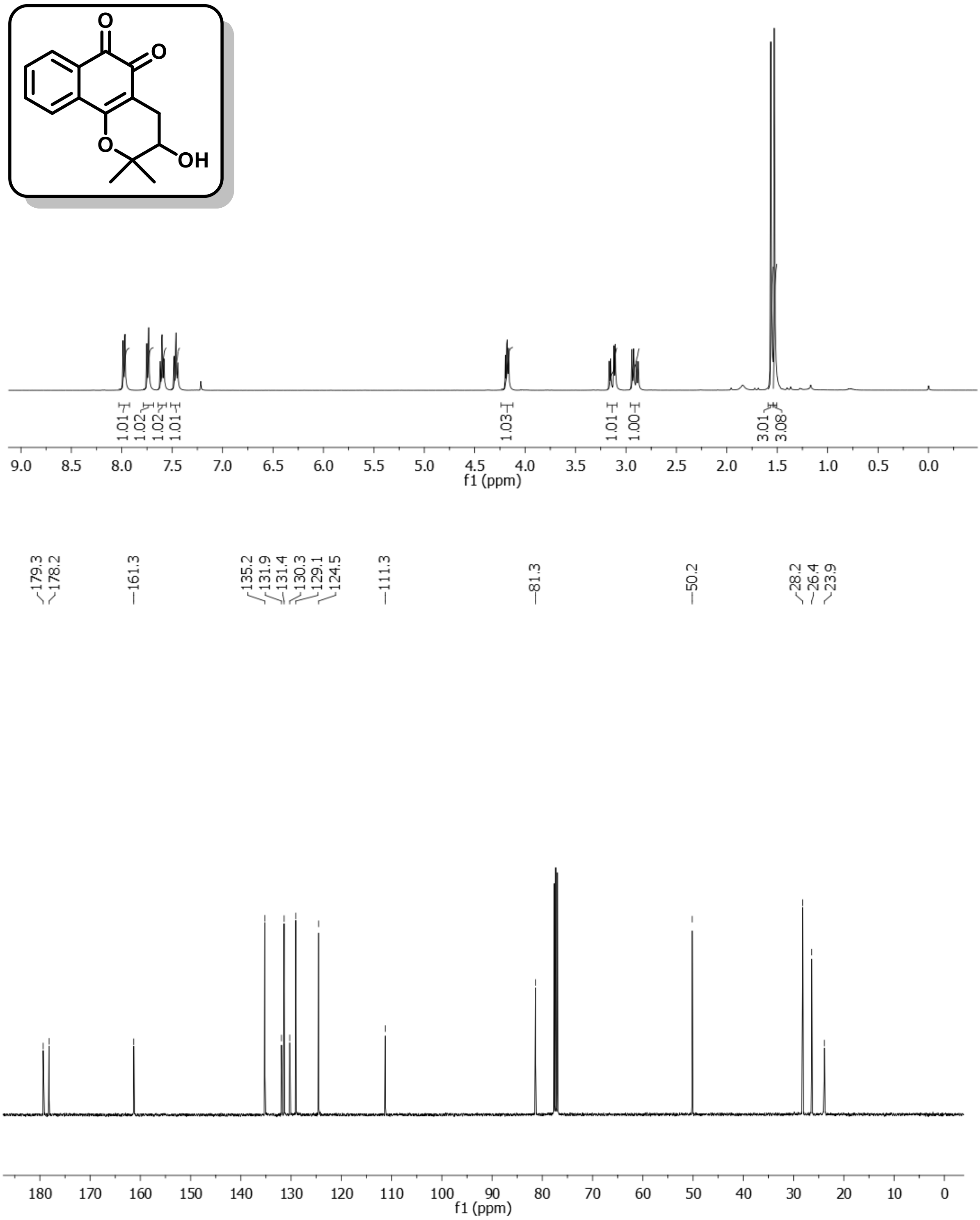

Figure S18. ^{1}H and ^{13}C NMR spectrum of 3-hydroxy-β-lapachone at 400 MHz in $CDCl_3$ (300 K).

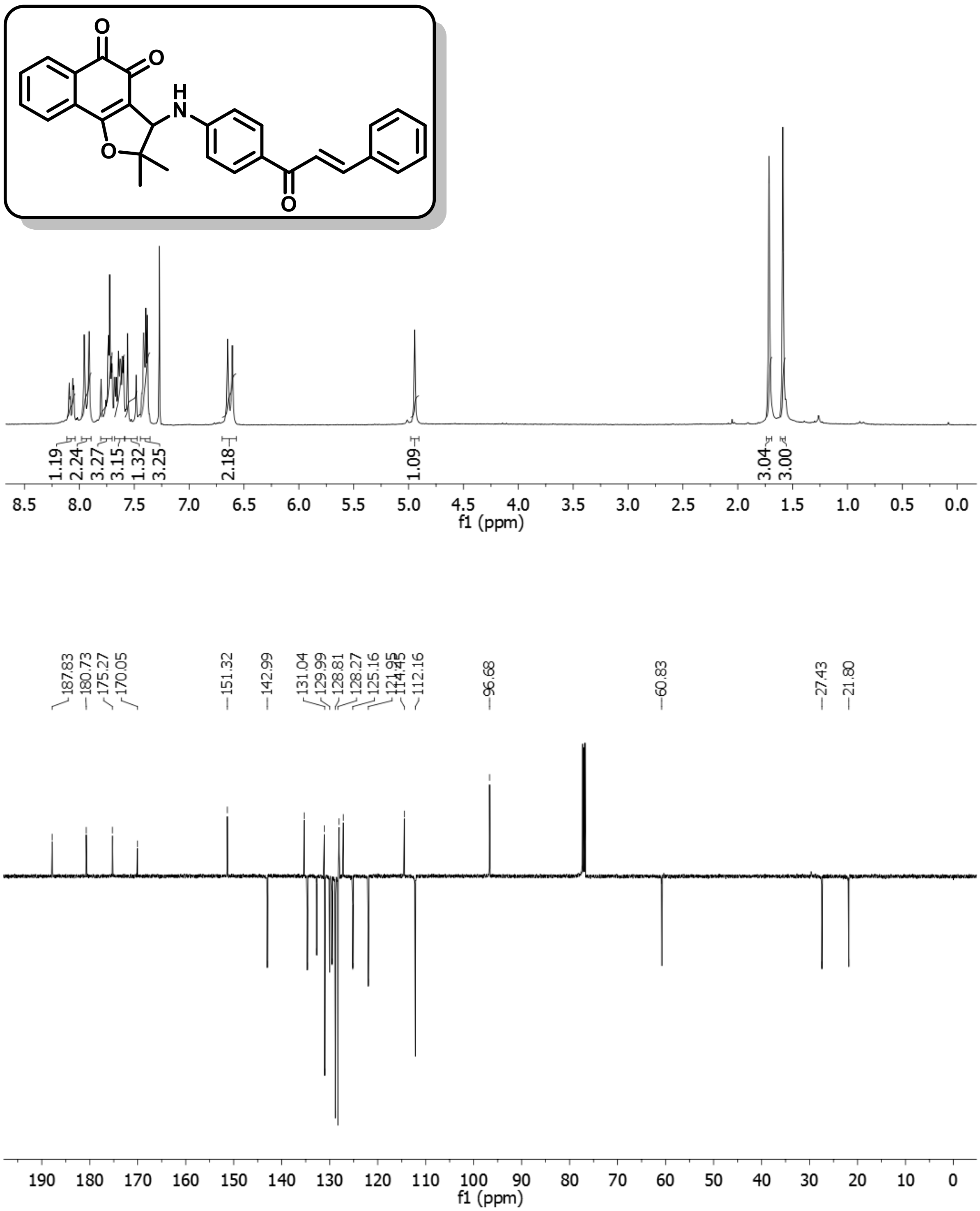

Figure S19. ^{1}H and DEPT spectra of nor-β-lapachone-based chalcone recorded in $CDCl_3$ (300 K).

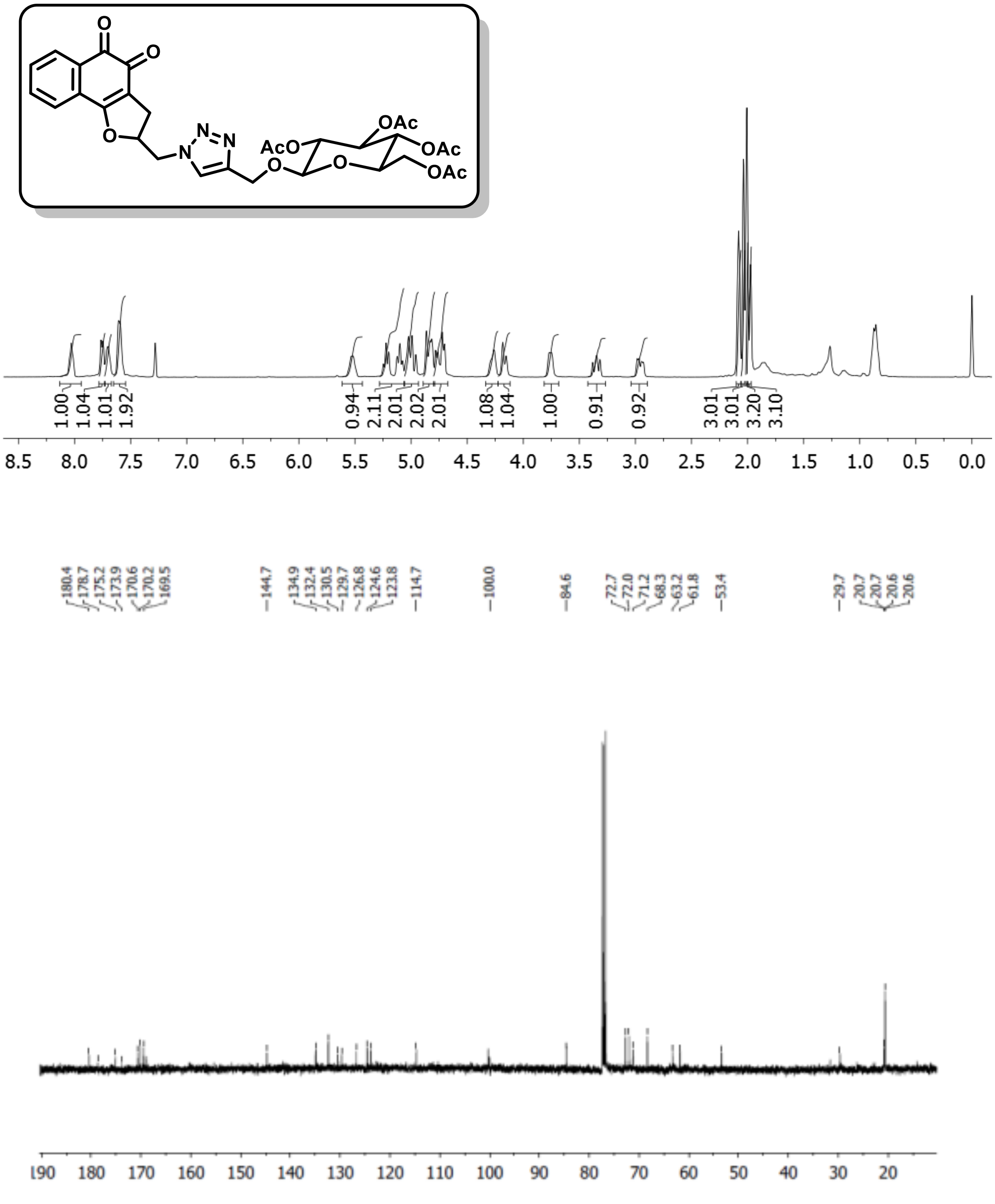

Figure S20. ^{1}H and ^{13}C NMR spectra of quinone-based 1,2,3-triazole-carbohydrate recorded in $CDCl_3$ (300 K).

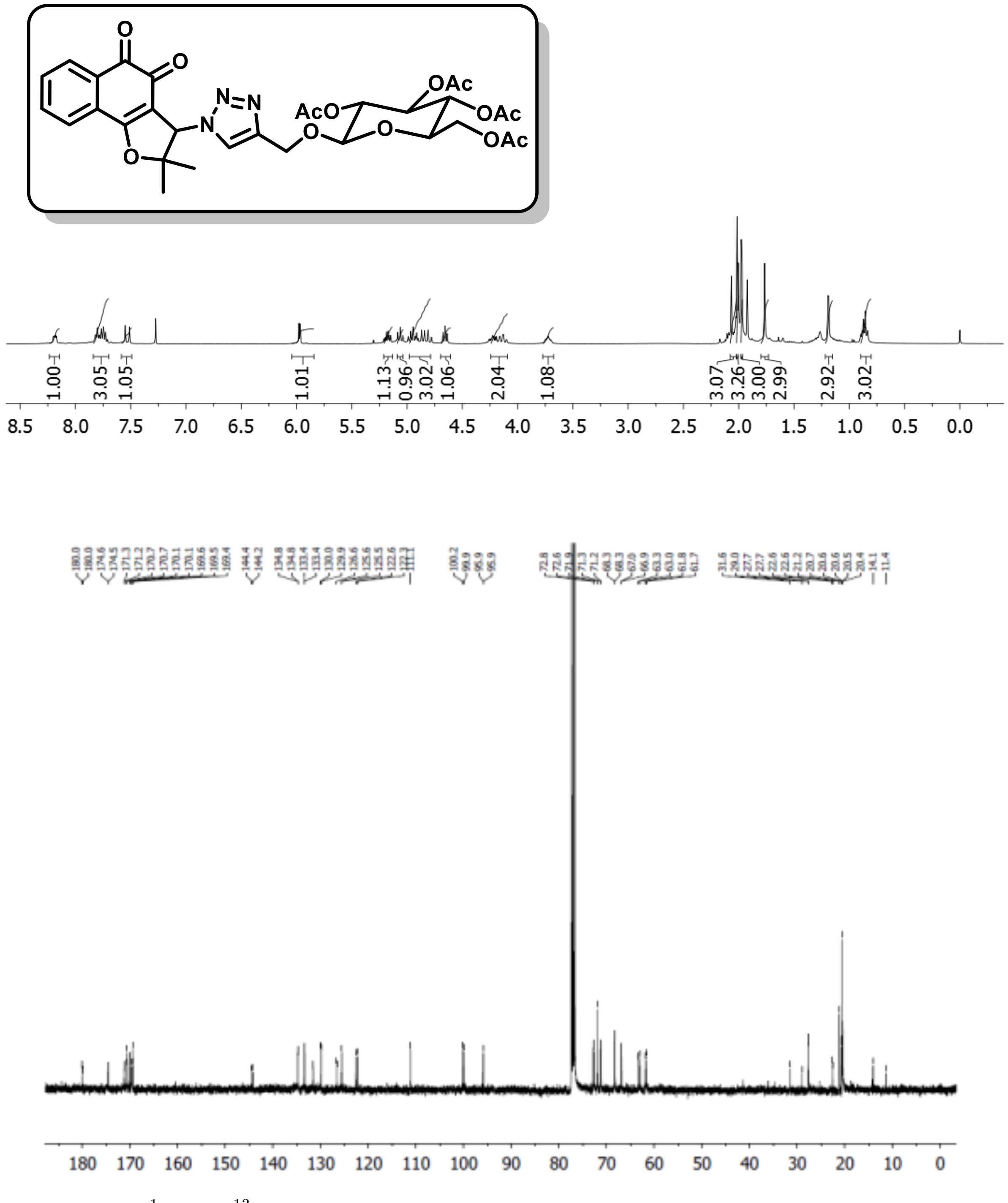

Figure S21. ^{1}H and ^{13}C NMR spectra of quinone-based 1,2,3-triazole-carbohydrate recorded in $CDCl_3$ (300 K).

2.9 Dynamic light scattering

Table S17. Dynamic light scattering data for β-lapachone and lapachol at varying concentrations in H_2O + 0.1% DMSO (25 °C).

**Analyte**	**Concentration / µM**	**Count rate (kcps)**
H_2O	n/a	45.0
Lapachol	100	882.6
	50	198.4
	25	250.6
β-lapachone	100	136.1
	50	47.6
	25	60.7

3. References

1. T. Sander, J. Freyss, M. von Korff and C. Rufener, *J. Chem. Inf. Model.*, 2015, **55**, 460-473.
2. D. Reker, T. Rodrigues, P. Schneider and G. Schneider, *Proc. Natl. Acad. Sci. U.S.A.*, 2014, **111**, 4067-4072.
3. T. Rodrigues, F. Sieglitz, V. J. Somovilla, P. M. Cal, A. Galione, F. Corzana and G. J. Bernardes, *Angew. Chem. Int. Ed.*, 2016, **55**, 11077-11081.
4. T. Rodrigues, D. Reker, P. Schneider and G. Schneider, *Nat. Chem.*, 2016, **8**, 531-541.
5. D. Reker, A. M. Perna, T. Rodrigues, P. Schneider, M. Reutlinger, B. Monch, A. Koeberle, C. Lamers, M. Gabler, H. Steinmetz, R. Muller, M. Schubert-Zsilavecz, O. Werz and G. Schneider, *Nat. Chem.*, 2014, **6**, 1072-1078.
6. T. Rodrigues, D. Reker, J. Kunze, P. Schneider and G. Schneider, *Angew. Chem. Int. Ed.*, 2015, **54**, 10516-10520.
7. P. Schneider and G. Schneider, *QSAR Comb. Sci.*, 2003, **22**, 713-718.
8. M. Reutlinger, C. P. Koch, D. Reker, N. Todoroff, P. Schneider, T. Rodrigues and G. Schneider, *Mol. Inf.*, 2013, **32**, 133-138.
9. M. J. Keiser, B. L. Roth, B. N. Armbruster, P. Ernsberger, J. J. Irwin and B. K. Shoichet, *Nat. Biotechnol.*, 2007, **25**, 197-206.
10. J. Nickel, B. O. Gohlke, J. Erehman, P. Banerjee, W. W. Rong, A. Goede, M. Dunkel and R. Preissner, *Nucleic Acids Res.*, 2014, **42**, W26-31.
11. N. C. Gilbert, Z. Rui, D. B. Neau, M. T. Waight, S. G. Bartlett, W. E. Boeglin, A. R. Brash and M. E. Newcomer, *FASEB J.*, 2012, **26**, 3222-3229.
12. T. Hanke, F. Dehm, S. Liening, S. D. Popella, J. Maczewsky, M. Pillong, J. Kunze, C. Weinigel, D. Barz, A. Kaiser, M. Wurglics, M. Lammerhofer, G. Schneider, L. Sautebin, M. Schubert-Zsilavecz and O. Werz, *J. Med. Chem.*, 2013, **56**, 9031-9044.
13. G. Jones, P. Willett and R. C. Glen, *J. Mol. Biol.*, 1995, **245**, 43-53.
14. S. C. Hooker, *J. Am. Chem. Soc.*, 1936, **58**, 1190-1197.
15. K. Schaffner-Sabba, K. H. Schmidt-Ruppin, W. Wehrli, A. R. Schuerch and J. W. Wasley, *J. Med. Chem.*, 1984, **27**, 990-994.
16. L. F. Fieser and M. Fieser, *J. Am. Chem. Soc.*, 1948, **70**, 3215-3222.
17. D. R. d. Rocha, K. Mota, I. M. C. B. d. Silva, V. F. Ferreira, S. B. Ferreira and F. d. C. d. Silva, *Tetrahedron*, 2014, **70**, 3266-3270.
18. M. Niehues, V. P. Barros, S. Emery Fda, M. Dias-Baruffi, M. Assis and N. P. Lopes, *Eur. J. Med. Chem.*, 2012, **54**, 804-812.
19. E. P. Sacau, A. Estevez-Braun, A. G. Ravelo, E. A. Ferro, H. Tokuda, T. Mukainaka and H. Nishino, *Bioorg. Med. Chem.*, 2003, **11**, 483-488.
20. E. N. da Silva Junior, M. C. de Souza, A. V. Pinto, C. Pinto Mdo, M. O. Goulart, F. W. Barros, C. Pessoa, L. V. Costa-Lotufo, R. C. Montenegro, M. O. de Moraes and V. F. Ferreira, *Bioorg. Med. Chem.*, 2007, **15**, 7035-7041.
21. E. N. da Silva, Jr., C. F. de Deus, B. C. Cavalcanti, C. Pessoa, L. V. Costa-Lotufo, R. C. Montenegro, M. O. de Moraes, C. Pinto Mdo, C. A. de Simone, V. F. Ferreira, M. O. Goulart, C. K. Andrade and A. V. Pinto, *J. Med. Chem.*, 2010, **53**, 504-508.

22. R. S. Silva, E. M. Costa, U. L. Trindade, D. V. Teixeira, C. Pinto Mde, G. L. Santos, V. R. Malta, C. A. De Simone, A. V. Pinto and S. L. de Castro, *Eur. J. Med. Chem.*, 2006, **41**, 526-530.
23. E. N. da Silva Junior, M. C. de Souza, M. C. Fernandes, R. F. Menna-Barreto, C. Pinto Mdo, F. de Assis Lopes, C. A. de Simone, C. K. Andrade, A. V. Pinto, V. F. Ferreira and S. L. de Castro, *Bioorg. Med. Chem.*, 2008, **16**, 5030-5038.
24. J. S. Sun, A. H. Geiser and B. Frydman, *Tetrahedron Lett.*, 1998, **39**, 8221-8224.
25. C. Salas, R. A. Tapia, K. Ciudad, V. Armstrong, M. Orellana, U. Kemmerling, J. Ferreira, J. D. Maya and A. Morello, *Bioorg. Med. Chem.*, 2008, **16**, 668-674.
26. G. A. M. Jardim, T. T. Guimarães, M. d. C. F. R. Pinto, B. C. Cavalcanti, K. M. d. Farias, C. Pessoa, C. C. Gatto, D. K. Nair, I. N. N. Namboothiri and E. N. d. S. Júnior, *Med. Chem. Commun.*, 2015, **6**, 120-130.
27. G. A. M. Jardim, W. J. Reis, M. F. Ribeiro, F. M. Ottoni, R. J. Alves, T. L. Silva, M. O. F. Goulart, A. L. Braga, R. F. S. Menna-Barreto, K. Salomão, S. L. d. Castro and E. N. d. S. Júnior, *RSC Advances*, 2015, **5**, 78047-78060.
28. T. Rodrigues, N. Hauser, D. Reker, M. Reutlinger, T. Wunderlin, J. Hamon, G. Koch and G. Schneider, *Angew. Chem. Int. Ed.*, 2015, **54**, 1551-1555.
29. M. D. Ungrin, L. M. Singh, R. Stocco, D. E. Sas and M. Abramovitz, *Anal. Biochem.*, 1999, **272**, 34-42.
30. R. J. Wilson, S. A. Rhodes, R. L. Wood, V. J. Shield, L. S. Noel, D. W. Gray and H. Giles, *Eur. J. Pharmacol.*, 2004, **501**, 49-58.
31. G. Asboth, S. Phaneuf, G. N. Europe-Finner, M. Toth and A. L. Bernal, *Endocrinology*, 1996, **137**, 2572-2579.
32. P. T. Phelps, J. C. Anthes and C. C. Correll, *Eur. J. Pharmacol.*, 2005, **513**, 57-66.
33. H. J. Behrendt, T. Germann, C. Gillen, H. Hatt and R. Jostock, *Br. J. Pharmacol.*, 2004, **141**, 737-745.
34. L. Fischer, D. Szellas, O. Radmark, D. Steinhilber and O. Werz, *FASEB J.*, 2003, **17**, 949-951.
35. D. Steinhilber, T. Herrmann and H. J. Roth, *J. Chromatogr.*, 1989, **493**, 361-366.
36. R. A. Pufahl, T. P. Kasten, R. Hills, J. K. Gierse, B. A. Reitz, R. A. Weinberg and J. L. Masferrer, *Anal. Biochem.*, 2007, **364**, 204-212.
37. N. B. Waslidge and D. J. Hayes, *Anal. Biochem.*, 1995, **231**, 354-358.
38. I. Kilty, A. Logan and P. J. Vickers, *Eur. J. Biochem.*, 1999, **266**, 83-93.
39. R. E. Weishaar, S. D. Burrows, D. C. Kobylarz, M. M. Quade and D. B. Evans, *Biochem. Pharmacol.*, 1986, **35**, 787-800.
40. G. Piechele, S. Colli, E. Tremoli and A. Maggi, *Pharmacol. Res.*, 1993, **27**, 53-60.
41. O. Roche, P. Schneider, J. Zuegge, W. Guba, M. Kansy, A. Alanine, K. Bleicher, F. Danel, E. M. Gutknecht, M. Rogers-Evans, W. Neidhart, H. Stalder, M. Dillon, E. Sjogren, N. Fotouhi, P. Gillespie, R. Goodnow, W. Harris, P. Jones, M. Taniguchi, S. Tsujii, W. von der Saal, G. Zimmermann and G. Schneider, *J. Med. Chem.*, 2002, **45**, 137-142.
42. J. B. Baell and G. A. Holloway, *J. Med. Chem.*, 2010, **53**, 2719-2740.
43. M. Pouliot and S. Jeanmart, *J. Med. Chem.*, 2016, **59**, 497-503.
44. J. B. Baell, *J. Nat. Prod.*, 2016, **79**, 616-628.

45. P. A. Clemons, N. E. Bodycombe, H. A. Carrinski, J. A. Wilson, A. F. Shamji, B. K. Wagner, A. N. Koehler and S. L. Schreiber, *Proc. Natl. Acad. Sci. U.S.A.*, 2010, **107**, 18787-18792.
46. E. Gilberg, M. Gutschow and J. Bajorath, *J. Med. Chem.*, 2018, **61**, 1276-1284.
47. J. B. Baell and J. W. M. Nissink, *ACS Chem. Biol.*, 2018, **13**, 36-44.
48. W. P. Walters, A. A. Murcko and M. A. Murcko, *Curr. Opin. Chem. Biol.*, 1999, **3**, 384-387.
49. C. J. Li, L. Averboukh and A. B. Pardee, *J. Biol. Chem.*, 1993, **268**, 22463-22468.
50. J. H. Lee, J. Cheong, Y. M. Park and Y. H. Choi, *Pharmacol. Res.*, 2005, **51**, 553-560.
51. J. A. Imlay, *Nat. Rev. Microbiol.*, 2013, **11**, 443-454.
52. L. F. Medina, P. F. Hertz, V. Stefani, J. A. Henriques, A. Zanotto-Filho and A. Brandelli, *Biochem. Cell Biol.*, 2006, **84**, 720-727.
53. M. C. Becerra and I. Albesa, *Biochem. Biophys. Res. Commun.*, 2002, **297**, 1003-1007.
54. M. Goswami, S. H. Mangoli and N. Jawali, *Antimicrob. Agents Chemother.*, 2006, **50**, 949-954.
55. F. Wippich, B. Bodenmiller, M. G. Trajkovska, S. Wanka, R. Aebersold and L. Pelkmans, *Cell*, 2013, **152**, 791-805.
